# Ultrathin quasi-2D amorphous carbon dielectric prepared from solution precursor for nanoelectronics

**DOI:** 10.1038/s44172-023-00141-9

**Published:** 2023-12-20

**Authors:** Fufei An, Congjun Wang, Viet Hung Pham, Albina Borisevich, Jiangchao Qian, Kaijun Yin, Saran Pidaparthy, Brian Robinson, Ang-Sheng Chou, Junseok Lee, Jennifer Weidman, Sittichai Natesakhawat, Han Wang, André Schleife, Jian-Min Zuo, Christopher Matranga, Qing Cao

**Affiliations:** 1 Department of Materials Science and Engineering, University of Illinois Urbana-Champaign, Urbana, IL USA; 2 National Energy Technology Laboratory (NETL), Pittsburgh, PA USA; 3 NETL Support Contractor, Pittsburgh, PA USA; 4 Center for Nanophase Materials Sciences, Oak Ridge National Laboratory, Oak Ridge, TN USA; 5 Corporate Research, Taiwan Semiconductor Manufacturing Company (TSMC), Hsinchu, Taiwan, ROC; 6 Seitz Materials Research Laboratory, University of Illinois Urbana-Champaign, Urbana, IL USA; 7 National Center for Supercomputing Applications, University of Illinois Urbana-Champaign, Urbana, IL USA; 8 Department of Electrical and Computer Engineering, University of Illinois Urbana-Champaign, Urbana, IL USA; 9 Department of Chemistry, University of Illinois Urbana-Champaign, Urbana, IL USA; 10 Holonyak Micro & Nanotechnology Laboratory, University of Illinois Urbana-Champaign, Urbana, IL USA

**Keywords:** Electrical and electronic engineering, Electronic devices, Two-dimensional materials

## Abstract

Materials keeping thickness in atomic scale but extending primarily in lateral dimensions offer properties attractive for many emerging applications. However, compared to crystalline counterparts, synthesis of atomically thin films in the highly disordered amorphous form, which avoids nonuniformity and defects associated with grain boundaries, is challenging due to their metastable nature. Here we present a scalable and solution-based strategy to prepare large-area, freestanding quasi-2D amorphous carbon nanomembranes with predominant sp^2^ bonding and thickness down to 1–2 atomic layers, from coal-derived carbon dots as precursors. These atomically thin amorphous carbon films are mechanically strong with modulus of 400 ± 100 GPa and demonstrate robust dielectric properties with high dielectric strength above 20 MV cm^−1^ and low leakage current density below 10^−4^ A cm^−2^ through a scaled thickness of three-atomic layers. They can be implemented as solution-deposited ultrathin gate dielectrics in transistors or ion-transport media in memristors, enabling exceptional device performance and spatiotemporal uniformity.

## Abstract (web summary)

Fufei An and colleagues report a solution-based strategy to prepare large-area, freestanding amorphous quasi-2D carbon nanomembranes from coal-derived carbon dots. Their mechanical and dielectric properties make these attractive materials for applications in nanoelectronic devices such as memristors and 2D-based transistors.

## Introduction

Crystalline two-dimensional (2D) semiconductors and semimetals, e.g., transition-metal chalcogenides, black phosphorus, and graphene, have drawn lots of interest from the aspects of both fundamental studies and practical applications^1,2^. However, the lack of appropriate accompanying insulators prevents them from achieving their full performance potential in electronic devices^3^. Integrating 2D channels with 3D bulk amorphous metal oxides in transistors generally leads to poor interfaces with high concentrations of traps and scattering centers, while 2D crystalline dielectrics, such as hexagonal-boron nitride (h-BN), are promising dielectrics for 2D transistors but suffer from large leakage current and spatial non-uniformity caused by crystalline grain boundaries, as well as fabrication difficulties and cost^4,5^. Low-dimensional amorphous dielectrics, as a counterpart of 3D SiO_2_ for bulk silicon, could be ideal for 2D-material-based nanoelectronics with their intrinsic ultrathinness and capability to maintain a smooth and homogenous 2D surface. However, synthesis of amorphous materials with thickness approaching the atomic limit is a substantial challenge since non-crystalline phases are thermodynamically unstable with the tendency to transform into crystalline phases under typical low-dimensional-nanomaterial growth conditions. Current approaches to prepare 2D amorphous films rely on the adoption of plasma-enhanced chemical-vapor deposition (CVD) or pulsed-laser deposition, where the power provided by the excited species in plasma or the energetic pulsed laser can lead to the formation of continuous films but is insufficient to promote their crystallization^6–9^. For both methods, the spatial uniformity and area coverage of the prepared 2D amorphous films could be limited, and especially the film thickness cannot be precisely controlled with atomic level of precision, in particular beyond the first monolayer when the reaction can no longer be catalyzed by the substrate. These limitations have so far prevented their adoption in functional application demonstrations which require films with large-area homogeneity and precisely tuneable thickness from monolayer to multilayers, ideally synthesized directly on device substate.

Here we report the wafer-scale synthesis of ultrathin quasi-2D amorphous carbon with thickness down to 1–2 atomic layers from solution-processable carbon-dot precursors directly on non-catalytic substrates. The prepared one layer of coalesced carbon dots is an atomically thin, mechanically strong amorphous film predominantly composed of sp^2^ carbon with low surface dangling bond density, and their few-layer assemblies are robust nanodielectrics with low leakage current density and high breakdown field strength. This synthesis strategy is distinct from vacuum depositions, with substantial advantages in enabling a solution-based process that is not only scalable but can be repeated in a layer-by-layer fashion for producing freestanding membranes from 1–2 atomic layers to multi-layered stacks with precisely controlled nanometer thickness. The achieved macroscopic uniformity, atomic-level thickness control, and wafer-scale processability allow engineered incorporation of prepared quasi-2D amorphous carbon films as dielectric in nanoelectronic devices, where their unique structure and properties were exploited to enable enhanced device performance and uniformity.

## Results and discussion

### Synthesis and characterizations of atomically thin quasi-2D amorphous carbon films

In this synthesis process, 2–4 nm diameter carbon dots are first derived from high-purity coal char and suspended in toluene after being functionalized with oleylamine (See Methods). The unique structure of coal char, which is composed of angstrom or nanometre-sized aromatic sp^2^ carbon domains linked with aliphatic carbon chains, allows the production of high-purity carbon dots with single atomic layer thickness of 0.42 nm, comparable to the interlayer distance in graphite of 0.33 nm, as measured by scanning tunneling microscopy (STM)^10^. Spin coating subsequently assembles these carbon dots into a quasi-monolayer where they are packed with random orientation as driven by the centrifugal force on a wafer substrate functionalized with hexamethyldisilazane (HMDS)^11^. There could be some inter-dot overlapping between the edges of neighboring carbon-dot precursors to afford a fully continuous film with 1–2 atomic layer thickness fluctuation. Annealing the assembly in an inert atmosphere at 500 ^o^C on non-catalytic substrates such as SiO_2_ then coalesces these densely packed carbon dots into a connected ultrathin film showing atomic-level roughness but without well-defined crystalline order, through the thermal-assisted cleavage of the amide bonds and the radical addition, followed by non-catalytic graphitization to seal most of the inter-dot gaps (See Methods). The continuous film produced can be suspended and transferred to different substrates for further characterizations.

**Fig. 1:**
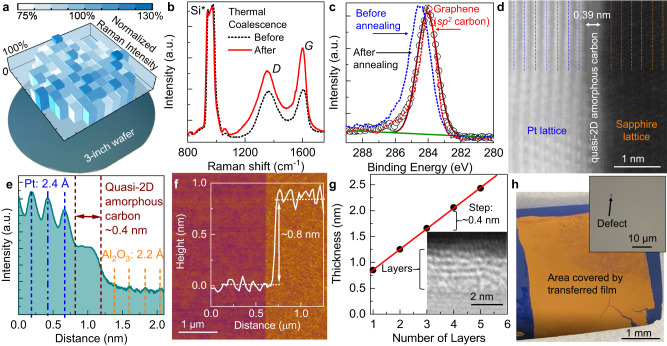
Wafer-scale ultrathin quasi-2D amorphous carbon prepared by the assembly and coalescence of coal-derived carbon dots. **a** Optical image of an atomically thin quasi-2D amorphous carbon film deposited on a 3-in. diameter SiO_2_/Si wafer with the normalized total Raman intensity for the D and G bands mapped over 96 points. The color code illustrates a Raman intensity range from 75% to 130% of the average with standard deviation of 10%. **b** Raman spectra of the carbon-dot assembly as deposited (black dotted line) and after coalescence (red solid line). Si* marks the second-order Raman scattering by optical phonons of silicon. **c** High-resolution C1*s* XPS spectrum of the ultrathin quasi-2D amorphous carbon formed on SiO_2_/Si substrate (black circles), with the fitted curve (brown solid line) showing a single carbon sp^2^ peak at 284.0 eV, while the film before annealing (blue dashed line) contains a substantial amount of sp^3^ carbon with a higher binding energy of 284.8 eV. XPS spectrum of crystalline graphene was measured as internal control to mark the position of sp^2^ carbon peak (red dashed line). Green solid line is the baseline subtracted before fitting. Reconstructed cross-sectional STEM image (part **d**) and associated contrast intensity profile (part **e**) of an ultrathin quasi-2D amorphous carbon film deposited on a *c*-cut sapphire wafer with a Pt protective layer on top. Blue and orange dashed lines serve as visual guide to mark the atomic planes in Pt and sapphire, respectively. Dark red dashed lines mark the material interfaces. **f** AFM image of ultrathin quasi-2D amorphous carbon film patterned to generate an edge by photolithography and oxygen reactive-ion etching (RIE). Inset: The averaged line cut showing a step height ~0.8 nm. **g** Film thickness as a function of the number of layers deposited. The red solid line is a linear fitting to the data. Inset: cross-sectional TEM micrograph showing the atomic planes. **h** Optical image of an ultrathin quasi-2D amorphous carbon membrane transferred to a substrate of Au (200 nm)/SiO_2_ (90 nm)/Si, after immersing in Au etchant. The area not covered by the transferred film appears as dark blue, while the intact Au region corresponds to the transferred quasi-2D amorphous carbon film. Inset: a magnified view showing an etched pit corresponding to a pin-hole defect in the transferred atomically thin nanomembrane.

This solution-based process allows the scalable preparation of amorphous carbon films with thickness down to 1–2 atomic layers covering 3-inch wafers (Fig. 1a). The macroscopic homogeneity of synthesized films is suggested by the uniform Raman intensity mapped across the entire wafer without systematic variations (also see Supplementary Note 1 and Supplementary Fig. 1). Comparing the Raman spectra of the as-deposited carbon-dot-precursor assembly and the ultrathin continuous film formed post-annealing shows a slight decrease of the D to G band ratio (*I*_D_/*I*_G_), indicating the structure transformation along with coalescence into a strong network of interconnected carbon atoms, while the average defect distance is still less than 1 nm with *I*_D_/*I*_G_ *=* 1.15 (Fig. 1b). Their high-resolution X-ray photoelectron spectroscopy (XPS) spectra reveal that the thermal crosslinking of the carbon dots, which initially contain a mixture of sp^2^ and sp^3^ hybridized carbon, forms a film predominately composed of sp^2^ carbon, similar to graphene (Fig. 1c), although there might be still some sp^3^ content left below the detection limit of XPS^12,13^. The high proportion of sp^2^ carbon with planar bonding helps to minimize the density of surface dangling bonds. This feature is quite different from CVD-deposited thin diamond-like carbon, which has substantial sp^3^ carbon and hydrogen contents.

The thickness of the prepared ultrathin quasi-2D amorphous carbon film was measured precisely first based on its high-resolution cross-sectional scanning transmission electron microscopy (STEM) images (Fig. 1d, see also Supplementary Note 2 and Supplementary Figs. 2–4 for comparison with controls and film uniformity). As-synthesized film was sandwiched between a crystalline sapphire substrate and a platinum capping layer. With the atomic planes of sapphire and platinum clearly visualized in the micrographs and manifested as peaks in the contrast intensity profile (Fig. 1e), the average thickness of the film over the 40–60 nm projection depth was determined as 0.41 ± 0.04 nm, which is comparable to the interlayer spacing of graphite and corresponding to a single atomic layer of carbon atoms. The atomic force microscopy (AFM) gave a thickness and surface roughness of about 0.8 nm and 0.1 nm, respectively, possibly indicating the presence of some two-atomic-layer thick regions (Fig. 1f). The smaller film thickness compared to the 2–4 nm diameter of carbon-dot precursors and the low surface roughness as measured by both AFM and STEM suggest that the spin-casting assembly aligns the basal plane of carbon dots parallel to the wafer surface, as assisted by the favorable interactions with HMDS^14^, and these precursors predominantly connect laterally, likely assisted by their overlapping edges, to form the quasi-2D ultrathin film with 1–2 atomic-layer thickness.

A unique attribute of this solution-based process is that it allows the formation of multilayered films in a continuous layer-by-layer fashion. After each deposition-coalescing cycle, the film thickness increased precisely by 0.4 nm, which is again comparable to the interlayer spacing of graphite, as measured by both AFM and transmission electron microscopy (TEM). The atomic planes parallel to the wafer surface, aligned through the attractive π–π interactions between the basal planes of the bottom layer and the carbon-dot precursors during assembly (similar to the interaction between HMDS and carbon dots in forming the base layer), can be clearly visualized in cross-sectional TEM/STEM, whose total counts agree with the number of deposition cycles performed (Fig. 1g, Supplementary Fig. 5). In some regions, the layered stacking is not clearly visible, because the film is amorphous and therefore the atoms are not perfectly flat, due to the presence of Stone-Wales defects and atomic-scale thickness fluctuations, in the projection depth. The smoothness of both monolayered and multilayered films suggested the absence of any bulk, i.e., bigger than atomic scale, residues or impurities.

With carbon-dot precursors covalently crosslinked together, the prepared ultrathin quasi-2D amorphous carbon film, even with a thickness down to merely 1–2 atomic layers, is mechanically robust enough to be transferred to other substrates as a complete and continuous membrane (See Methods), and the transferred atomically thin film can serve as a chemical etching mask. The quantitative pinhole-type defect density of the transferred film was determined by wet-etching based chemical amplification as 17 ± 5 × 10^3^ defects per mm^2^ (Fig. 1h, Supplementary Note 3 and Supplementary Fig. 6)^15^. Several factors contribute to the pin-hole defects, such as particles on the substrate or in the precursor solution, and especially the mechanical damages to the film during transfer. The fact that the defect density is much lower than the density of boundaries among carbon dots after assembly indicates that these carbon dots not only coalesce into a homogenous film after annealing but the crosslinking mechanism tends to eliminate the inter-particle geometric gaps^16^.

**Fig. 2:**
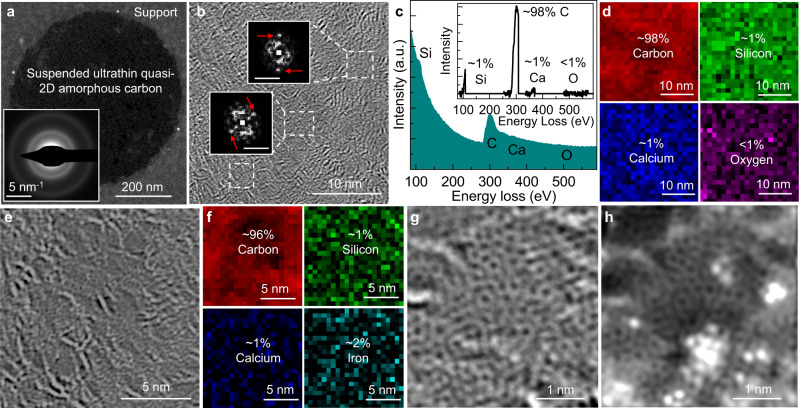
Electron microscopy characterizations of the freestanding atomically thin quasi-2D amorphous carbon nanomembrane. **a** Low-magnification ADF-STEM image and SAED patterns (inset) of a freestanding ultrathin quasi-2D amorphous carbon membrane suspended over the holes of a holey-carbon grid. Medium-resolution STEM image (**b**), associated EELS spectrum (**c**, inset: spectrum after background subtraction), and the spatial mapping of elements identified (**d**). In (**b**), white dashed lines mark the boundaries of regions showing regular atomic arrangement, with insets showing the corresponding fast Fourier transforms (scale bars: 5 nm^−1^). Red arrows point at the Bragg spots. High-resolution STEM image (**e**) and elemental mapping (**f**). Atomic-resolution aberration-corrected bright-field (**g**) and ADF (**h**) STEM images.

The transferred atomically thin (0.4–0.8 nm thick) quasi-2D amorphous carbon can be suspended as freestanding nanomembranes on TEM grid for imaging (Supplementary Fig. 7). Note here that the as-deposited carbon-dot assembly or those processed at temperature below 400 ^o^C cannot be suspended over these holey-carbon cavities, indicating that the covalent-like bond formation induced by the 500 ^o^C annealing is critical to ensuring the formation of continuous and robust quasi-monolayers. The uniform contrast throughout in the low-magnification annular dark-field (ADF)-STEM image confirms that the film is macroscopically homogenous, and the selected-area electron diffraction (SAED) patterns show a characteristic diffuse halo, which verifies its amorphous nature (Fig. 2a). Under higher magnification, some randomly oriented domains with partially regular lattice can be visualized, where the localized fast Fourier transforms exhibit Bragg spots (Fig. 2b). The presence of brighter ridges indicates the atomic-level roughness generated during the assembly of carbon dots and preserved after their coalescence. The corresponding electron-energy-loss spectroscopy (EELS) spectrum suggests that the film prepared is predominantly composed of carbon with quite a low abundance of silicon, calcium, oxygen, and possibly iron impurities (Fig. 2c and Supplementary Fig. 8), which distribute uniformly and are likely coming from environmental contaminations (Fig. 2d). Higher-resolution STEM images (Fig. 2e) further reveal the regular patterns of carbon atoms (EELS mapping in Fig. 2f) in some areas, surrounded by regions of lower order with flexible and continuous atomic structures similar to what is described in the modern crystallite theory of amorphous solids instead of sharp grain boundaries as what would be expected in a nanocrystalline film^6,17^. Aberration-corrected high-resolution STEM was then used to examine the arrangement of carbon atoms. The obtained bright-field (Fig. 2g) and ADF (Fig. 2h) images show the distorted structure composed of carbon rings with different sizes and orientations, with bright spots likely caused by the presence of some mobile metal impurity atoms on the surface, which are also visible in the EELS spectra. Such amorphous structures lacking long-range order and featuring the likely co-existence of carbon rings with various sizes and orientations, are uniformly present over large area (Supplementary Fig. 9). Note here that the low operation voltage of 60 kV and the ultrahigh vacuum chamber of Nion microscope minimized the radiation damages to atomically thin carbon films during imaging. Meanwhile, we acknowledge that the blurring of the atomic positions and the density variations across the regions in these STEM images suggest the presence of some bilayer regions, with the associated moiré effects distorting the contrast.

### Physical properties of quasi-2D amorphous carbon prepared from solution precursors

**Fig. 3:**
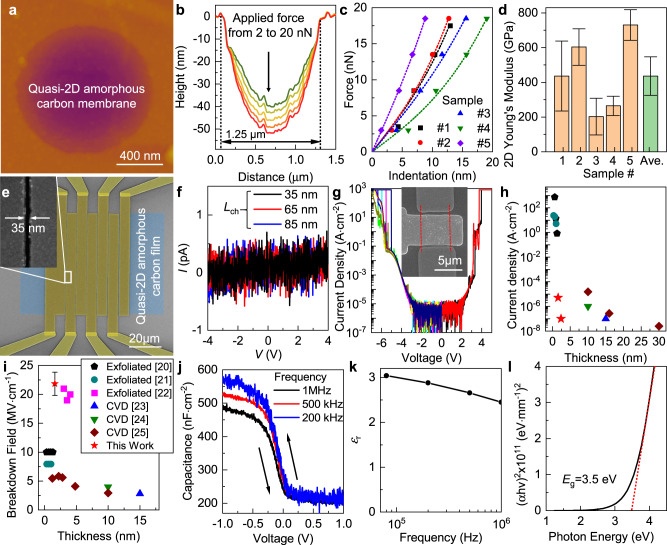
Physical properties of quasi-2D amorphous carbon prepared from carbon-dot precursors. **a** AFM scan of an ultrathin (1–2 atomic-layer thick) quasi-2D amorphous carbon nanomembrane suspended over a 1.25 μm diameter well. **b** Height profiles from multiple indentations with the force applied increasing from 2 to 5, 10, 15, and 20 nN from top to bottom, colored from green to red. Force-displacement curves (**c**, symbols: experimental data; dashed lines: fitting to the linear elastic deformation expression) measured from multiple (five, each represented with a different color) suspended ultrathin membranes, and their extracted 2D Young’s moduli (**d**, orange represents each sample and green represents the average; error bars represent uncertainties of fitted parameters). **e** False-colored SEM image of an array of two-terminal devices with 50 μm channel width (*W*) to measure the lateral resistance of quasi-2D amorphous carbon. Inset: SEM micrograph showing the smallest *L*_ch_ down to 35 nm. **f** Current (*I*)-voltage (*V*) curves measured for two-terminal devices shown in (**e**) with *L*_ch_ of 35 nm (black), 65 nm (red), and 85 nm (blue), respectively. **g** Current density flowing vertically across triple-layered quasi-2D amorphous carbon films (1.2–1.6 nm thick) sandwiched between 5 μm-wide graphene bottom electrodes and 5 μm-wide metal top electrodes. Results from ten devices biased with both polarities were plotted together with each represented by a different color. Inset: SEM micrograph of the graphene/quasi-2D amorphous carbon trilayer/metal capacitor where red dashed lines serve as visual guide to mark the boundary of the graphene bottom electrode. Comparison of the leakage current density (**h**) and the dielectric breakdown field (**i**) of quasi-2D amorphous carbon films and h-BN prepared with both mechanical exfoliation and CVD. The leakage current through the exfoliated h-BN and the associated breakdown field in ref. ^21^ and ref. ^22^ were determined in a single grain using the conductive-AFM with a circular tip-sample contact area with diameter of merely 25 ± 10 nm and 2–4 nm, respectively. **j** Capacitance-voltage characteristics of a disk-shaped 20 μm-diameter metal/quasi-2D amorphous carbon film (10 sequentially deposited layers, ~4.5 nm thick)/silicon capacitor, measured under different frequencies from 200 kHz (blue), 500 kHz (red), to 1 MHz (black). Arrows mark the voltage sweeping directions during the measurement. **k** Frequency dependence of *ε*_r_ of quasi-2D amorphous carbon film evaluated between 10^4^ and 10^6^ Hz. **l** Tauc plot with the extrapolation of the linear region (red dashed line) to determine the effective optical bandgap (*E*_g_) of the ultrathin quasi-2D amorphous carbon.

Structure-wise, the continuous and uniform carbon films synthesized from solution-deposited carbon-dot precursors exhibit thickness approaching the atomic limit (1–2 atomic layers) with predominant sp^2^ bonding and highly disordered amorphous atomic structures. These attributes lead to their exceptional physical properties as a mechanically strong and electrically robust ultrathin dielectric. Their mechanical properties were first characterized by AFM nanoindentation performed on atomically thin (0.4–0.8 nm thick) nanomembranes suspended over 1.25 μm wide and 3 μm deep circular holes (Fig. 3a)^18^. AFM topography was measured by contact mode with the displacement under the applied force (*F*) determined from the center of the membranes (Fig. 3b). The 2D elastic constant was then calculated by fitting the force-displacement curves using the equation of $F=\frac{2\pi \sigma \delta }{{{{{\mathrm{ln}}}}}(\alpha /r)}+\frac{E{q}^{3}{\delta }^{3}}{{\alpha }^{2}}$, where α and r are the radii of the suspended film and the AFM tip; *σ*, *δ*, and *E* are the 2D pretension, deflection, and 2D Young’s modulus, respectively; and *q* *=* 1.02 is obtained based on the Poisson’s ratio of the basal plane of graphite of 0.165 (Fig. 3c)^19^. The extracted high Young’s modulus of 400 ± 100 GPa, which is comparable to that of crystalline graphene and h-BN but higher than that of 2D amorphous carbon monolayer grown by CVD, further verifies the homogeneity and continuity of the ultrathin quasi-2D amorphous carbon prepared from the assembly and coalescence of carbon dots with strong lateral connections, which are very likely of covalent nature (Fig. 3d).

The electrical conductivity of the synthesized quasi-2D amorphous carbon films was first evaluated in the lateral (in-plane) direction (Fig. 3e). Even with a channel length (*L*_ch_) down to 35 nm, the measured current flowing laterally along the ultrathin quasi-2D amorphous carbon was still less than the detection limit of our measurement setup of 0.5 pA (Fig. 3f), giving a lower bound of the two-terminal resistance of 10 TΩ and resistivity above 1 × 10^9^ Ω cm, which is comparable to that of h-BN thin films but much higher than that of CVD-grown 2D amorphous carbon which contains a high density of embedded nanocrystallites facilitating the hopping-based carrier transport^6–9^. The vertical (out-of-plane) conductivity was then characterized as the leakage current flowing through a metal–insulator–semimetal capacitor, where the on-chip synthesized quasi-2D amorphous carbon film separated a graphene bottom and a metal top electrode. A very low leakage current density below 10^−4^ A cm^−2^ was obtained across a thin film composed of three consecutively deposited layers of ultrathin quasi-2D amorphous carbon to afford an overall thickness of merely 1.2–1.6 nm (Fig. 3g). Compared to exfoliated h-BN with similar thickness^4,20–25^, this leakage current density is over 1,000 times lower (Fig. 3h), likely assisted by the quantum mechanical destructive interference between different pathways for wave functions of electrons to tunnel through a barrier with atomic-scale potential energy variation^26^, and the absence of grain boundaries. The leakage current increased abruptly above −5.5/ + 3.5 V, leading to electric breakdown with a breakdown field above 20 MV cm^-1^, which is much higher than that of diamond^27^. It is because electrons passing through quasi-2D amorphous carbon films have a higher probability to be slowed down by scattering. Therefore, a higher electric field must be applied to induce breakdown by impact ionization^28^. The breakdown strength of quasi-2D amorphous carbon films is also higher than that of crystalline or amorphous BN^7^ (Fig. 3i), evaluated in the commonly adopted graphene/dielectric/metal configuration where the orders of magnitude higher conductivity of metal and graphene compared to the dielectric films ensures that the applied electric field falls entirely across the dielectric sandwiched in between and the ultrathin thickness of graphene ensures that there is no field focusing effect at the corners of the bottom electrode^20,23^. The improvement is especially substantial (>4 times higher) compared to large-area h-BN grown by CVD^23–25^.

The relative dielectric constant (*ε*_r_) of the quasi-2D amorphous carbon was determined from the capacitance-voltage curves of metal-insulator-semiconductor capacitors constructed with 10 stacked layers of consecutively deposited films (total thickness ~4.5 nm) sandwiched between a p-doped silicon wafer and Au top electrode (Fig. 3j). No hysteresis was observed, which is indicative of a high-quality interface formed between silicon and the dangling-bond-free quasi-2D amorphous carbon film with predominant sp^2^ bonding. The extracted *ε*_r_ was 3 at 100 kHz, which is comparable to the out-of-plane dielectric constant of h-BN^29^, and reduced slightly with the increase of frequency (Fig. 3k). It enables an equivalent oxide thickness (EOT), which is the thickness of silicon oxide film that provides the same capacitance, of ~2 nm for a *~* 1.6 nm thick quasi-2D amorphous carbon trilayer. To enable an EOT below 1 nm, the *ε*_r_ of quasi-2D amorphous carbon needs to be increased, potentially through introducing heteroatoms such as chlorine, which can form covalent bonds with carbon with high permanent dipole moment and polarizability, as dopants^30^. An effective optical bandgap ~3.5 eV was extracted by fitting the Tauc plot where the absorption coefficient was determined via ellipsometry (Fig. 3l). These measured properties of solution-prepared atomically-thin quasi-2D amorphous carbon are distinct from graphene, but they agree with what can be expected from the electronic structures of 2D amorphous carbon calculated using density-functional theory (DFT): In the amorphous structure, electronic states near the Fermi level are strongly localized, and this leads to both low carrier-transmission probability and the existence of an effective optical bandgap (Supplementary Note 4 and Supplementary Fig. 10).

### Solution-deposited quasi-2D amorphous carbon as critical dielectrics in nanoelectronics

Their intrinsic ultrathinness as determined from cross-sectional STEM/TEM and AFM, amorphous structure composed of heterogeneous carbon rings without sharp crystalline grain boundaries as evident from SAED/FFT patterns and top-view STEM, smooth sp^2^ carbon surface free of high-density dangling bonds as suggested by XPS, their robust mechanical and dielectric properties verified directly in nanoindentation and electrical measurements (Fig. 3h, i, and see also Supplementary Note 5 and Supplementary Table 1, for comparison with h-BN), together with scalable solution-based on-chip synthesis process and precise thickness control with atomic-scale resolution in continuous layer-by-layer deposition, make wafer-scale synthesized ultrathin quasi-2D amorphous carbon films hold great promise as dielectrics to enable nanoelectronic devices with enhanced performance at lower cost.

**Fig. 4:**
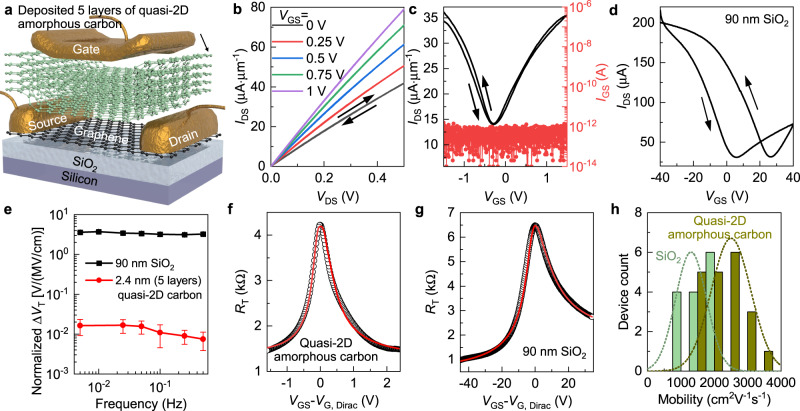
Application of quasi-2D amorphous carbon films as the gate dielectric in graphene transistors. **a** Schematic of a graphene transistor employing 5 sequentially deposited layers of quasi-2D amorphous carbon as top-gate dielectric. **b** Output characteristics. *L*_ch_ and *W* are 3.5 μm. Arrows indicate the source-drain voltage (*V*_DS_) sweeping directions. The gate voltage (*V*_GS_) increases from 0 V to 1 V from bottom to top at a step of 0.25 V. *I*_DS_: source-drain current. **c** Device transfer characteristics (left axis, black line) and the associated gate-leakage current (*I*_GS_, right axis, red dots). Arrows indicate the *V*_GS_ sweeping directions. Applied *V*_DS_ is 0.2 V. **d** Transfer characteristics of bottom-gated graphene transistors employing 90 nm SiO_2_ as the gate dielectric. The device *L*_ch_ and *W* are all 3.5 μm. Applied *V*_DS_ is 0.2 V. **e** Hysteresis width normalized with the gate dielectric field (maximum of *V*_GS_/dielectric thickness) as a function of the sweep frequency, which correlates with the time required to complete one up-sweep and down-sweep cycle, for graphene transistors built with either 90 nm SiO_2_ (black) or five layers of atomically thin quasi-2D amorphous carbon (red) as the gate dielectrics. Error bars represent standard deviation. Experimental plot (black circles) and modeling fitting (red solid line) of the change of *R*_T_ as a function of the gate overdrive (*V*_GS_–*V*_G, Dirac_) for graphene transistors built with either quasi-2D amorphous carbon (**f**) or 90 nm SiO_2_ (**g**) as the gate dielectrics. **h** Comparison of the effective field-effect mobility *µ* of bottom-gated graphene transistors with SiO_2_ gate oxide (green) and top-gated devices with quasi-2D amorphous carbon as gate dielectric (dark yellow). Dotted lines are Gaussian fits to the data.

First, quasi-2D amorphous carbon films, as a counterpart of 3D SiO_2_ for bulk silicon, could be ideal for 2D-material, e.g., crystalline graphene, based nanoelectronics with their intrinsic ultrathinness and capability to maintain a smooth and homogenous surface with low surface dangling bond density. Five layers of sequentially deposited ultrathin quasi-2D amorphous carbon (total thickness ~2.4 nm) were first incorporated as top-gate dielectrics for graphene transistors (Fig. 4a, Methods). The device delivered a large on-state conductance above 250 μS μm^−1^ (Fig. 4b). The high capacitance provided by the nanometre-thick quasi-2D amorphous carbon film leads to enhanced electrostatic coupling between the metal gate and the graphene channel, in comparison to the bottom-gated devices with 90 nm SiO_2_ as the gate oxide, which reduced the required gate-voltage swing for full device operation by over one order of magnitude, together with >10 times improvement of the device transconductance (Fig. 4c, d). Moreover, the device hysteresis was reduced from ~20 V to 0.08 V even when operated under a higher gate electric field, likely due to the elimination of the dangling bonds associated with each layer of the deposited ultrathin quasi-2D amorphous carbon and thus the charge traps both inside the dielectric and at its interface with the graphene channel (Fig. 4e)^31^. Even with a drastically smaller dielectric-layer thickness, the device gate leakage current was still less than the detection/noise limit of ~1 pA over the entire *V*_GS_ range to minimize the device power consumption in off-state. The effective mobility *μ* of graphene transistors was extracted by fitting the resistance profiles to ${R}_{T}={R}_{c}+\frac{{L}_{ch}}{We\mu \sqrt{{n}_{0}^{2}+{n}^{2}}}$ (Fig. 4f, g), where *R*_T_ is the total resistance, *R*_c_ is the contact resistance, e is the elementary charge, and n_0_ is the residual carrier concentration^32^. *n* is the carrier concentration as ${V}_{GS}-{V}_{G,Dirac}=\frac{e}{{C}_{G}}n+\frac{\hslash {v}_{F}\sqrt{\pi n}}{e}$, where V_G,Dirac_ is the Dirac point, C_G_ is the gate dielectric capacitance, ℏ is the reduced Planck constant, and *v*_F_ ∼ 10^8^ cm s^−1^ is the Fermi velocity of carriers in graphene. With the *R*_c_ and *n*_0_ separately verified by the transmission-line method and capacitance-voltage measurements (Supplementary Note 6, Supplementary Figs. 11 and 12), it was confirmed that the mobilities extracted from top-gated graphene transistors adopting the quasi-2D amorphous carbon film as gate dielectric were on average twice as high as those of back-gated devices employing SiO_2_ (Fig. 4h). The higher μ is likely assisted by the absence of traps and scattering centers associated with dangling bonds at the graphene/dielectric interface^33,34^. The concurrent achievements of low leakage current density, reduced hysteresis, large gate capacitance, improved μ, and simple solution-based and on-chip deposition demonstrate the performance and processing advantages of quasi-2D amorphous carbon film as “native” low-dimensional amorphous gate dielectrics for graphene transistors, compared to either bulk metal oxides or polycrystalline 2D h-BN (See Supplementary Note 7 and Supplementary Table 2 for direct comparison).

**Fig. 5:**
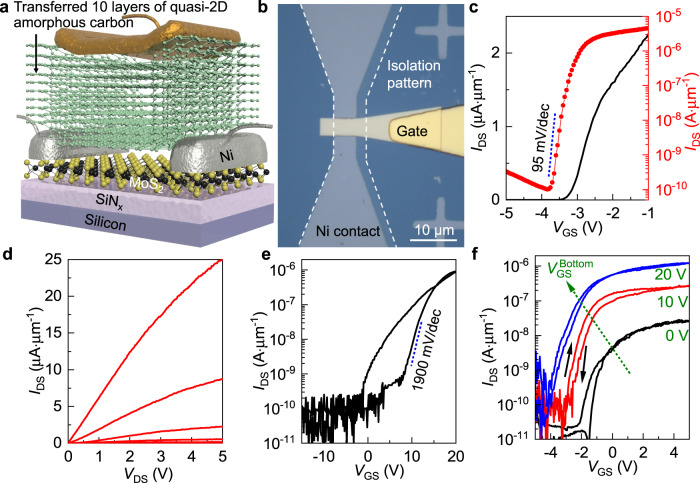
Application of quasi-2D amorphous carbon films as the gate dielectric in MoS_2_ transistors. Schematic (**a**) and optical micrograph (**b**, the white dashed line marks the boundary of monolayer MoS_2_ after device isolation) of a MoS_2_ transistor employing transferred 10 layers of quasi-2D amorphous carbon as top-gate dielectric. **c** Device transfer characteristics plotted in linear (left axis, black line) and logarithmic (right axis, red line and dots) scales. *L*_ch_ and *W* are 2 μm. Applied *V*_DS_ is 0.5 V and the back-gate bias is kept at 20 V, which modulates the MoS_2_ beneath the Ni electrodes to ensure ohmic contact. Blue dotted line marks subthreshold swing. **d** Device output characteristics. **e** Transfer characteristics of the same device measured with the top gate grounded and the bottom-gate bias varied from −15 V to 20 V. Applied *V*_DS_ is 0.5 V. **f** Transfer curves modulated by the top gate with back-gate bias (V_GS_^Bottom^) of 0 V (black), 10 V (red), and 20 V (blue), respectively. Applied *V*_DS_ is 0.5 V.

To further demonstrate their versatile applicability, quasi-2D amorphous carbon films were also integrated with monolayer MoS_2_ (See Methods, Supplementary Note 8, and Supplementary Fig. 13) as part of the device gate stack to realize high-performance 2D semiconductor transistors. Here ten layers of sequentially deposited ultrathin quasi-2D amorphous carbon with a total thickness of 4.5 nm were transferred on top of the MoS_2_ channel at room temperature to preserve the thermally unstable interface between MoS_2_ and Ni source-drain contacts (Fig. 5a, b). The transistor can be effectively switched by applying top-gate voltage range of 4 V (−5 to −1 V), giving an effective mobility above 15 cm^2^ V^−1^ s^−1^ and subthreshold swing less than 95 mV dec^−1^ (Fig. 5c). The nearly linear output characteristics at small source-drain bias indicate that the ohmic Ni-MoS_2_ contact was not affected (Fig. 5d). Compared to the operation of the same device but using the 100 nm Si_3_N_4_ as the back-gate dielectric, which requires a large gate-voltage range above 30 V and gives a lower effective mobility of 9 cm^2^ V^−1^ s^−1^ with subthreshold swing above 1900 mV dec^−1^ (Fig. 5e), the adoption of the nanometre-thick quasi-2D amorphous carbon film as the gate dielectric again leads to drastically improved device performances, in terms of the device transconductance, mobility, subthreshold swing, and hysteresis, similar to what was observed in graphene transistors (since the focus here is the dielectrics and there was substantial spatial variation in transferred CVD monolayer MoS_2_, we employed the same MoS_2_ channel with identical distribution of sulfur vacancy defects for fair comparison). It is likely that the field-effect mobilities and subthreshold swing of the demonstrated transistors are limited by the intrinsic CVD MoS_2_ quality or the barriers at the MoS_2_–Ni contacts. Nevertheless, these device characteristics compare favorably against the monolayer MoS_2_ transistors employing h-BN, which typically require much thicker film to suppress the leakage current density, or high-κ oxides, which typically do not provide the clean van der Waals interface for performance enhancement over SiO_2_/Si_3_N_4_ (see Supplementary Note 9 and Supplementary Table 3 for direct comparisons). The device hysteresis is small, and the top-gate transfer characteristics can be tuned by back-gate biasing, with the slope of the threshold voltage shift versus back gate correlated with the ratio of the bottom-gate to top-gate capacitance (Fig. 5f).

**Fig. 6:**
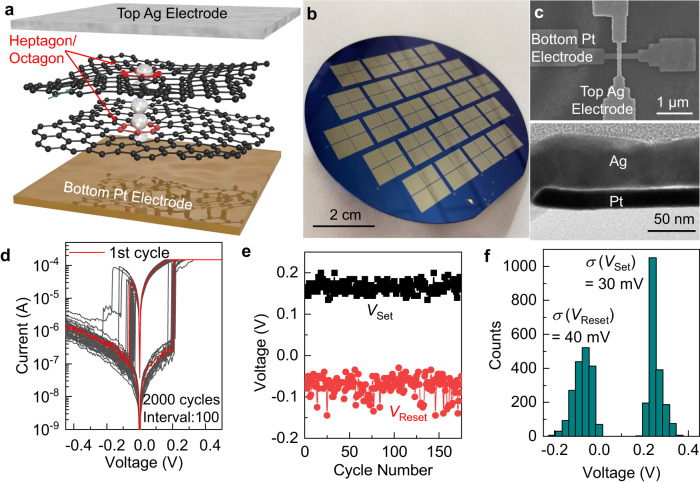
Application of quasi-2D amorphous carbon bilayers as the switching media in memristors to enable small cycle-to-cycle variability. **a** Exploded view of a memristor with a quasi-2D amorphous carbon bilayer as the ion-transport medium, where the silver atomic filament is confined within the large-size carbon rings (highlighted as red). **b** Optical image of a 3-in. wafer with the fabricated Pt/quasi-2D amorphous carbon bilayer/Ag memristor arrays. **c** Top-view SEM micrograph (top frame) and cross-sectional TEM image (bottom frame) of a completed device. **d** Current–voltage characteristics of a Pt/quasi-2D amorphous carbon bilayer/Ag memristor measured in 2000 consecutive cycles, with the first set/reset curve marked as red and the subsequent ones, with interval of every 100 cycles, plotted as gray solid lines. Set (*V*_set_, black) and reset (*V*_reset_, red) voltage variations over the first 175 *I*–*V* sweeps (**e**) and the histogram showing the distribution of *V*_set_ and *V*_reset_ in the entire 2000 cycles (**f**).

**Fig. 7:**
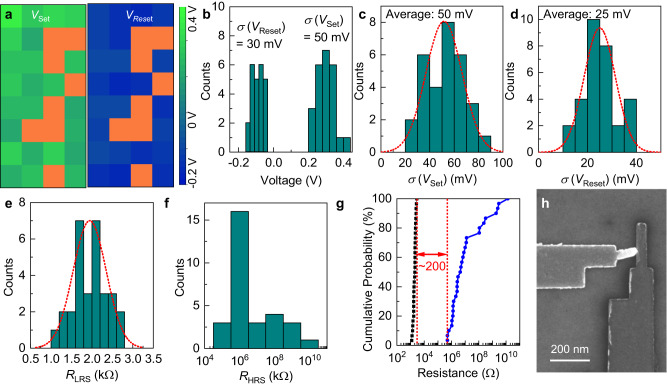
Device-to-device variability of memristors employing quasi-2D amorphous carbon bilayers as the ion-transport media. **a** Mapping of both the averaged *V*_set_ (left frame) and *V*_reset_ (right frame) of 32 Pt/quasi-2D amorphous carbon bilayer/Ag memristors in a 4 by 8 array (Color scale changes from green to blue corresponding to the change of voltage from 0.4 V to -0.2 V. Orange: failed devices without reliable resistive switching behavior.) **b** Histogram for the spatial distributions of *V*_set_ and *V*_reset_ shown in (**a**). Histograms showing the distribution of the standard deviation of *V*_set_ (**c**) and *V*_reset_ (**d**) extracted from 30 devices. Histograms showing the distribution of their low-resistance state (LRS) resistance (*R*_LRS_, **e**) and high-resistance state (HRS) resistance (*R*_HRS_, **f**) averaged over 25 cycles. Red dotted lines are Gaussian fits to the data. **g** Cumulative probability distribution of *R*_LRS_ (black squares) and *R*_HRS_ (blue circles) based on multiple switch cycles performed on multiple devices. The red dotted lines serve as a visual guide to mark an on/off ratio of at least 200 in the worst-case scenario. **h** SEM micrograph showing a failed device caused by the delamination of the Ag top electrode.

The intrinsic atomic-level thickness and the presence of large-size carbon rings inside the atomic structure of quasi-2D amorphous carbon also make its multilayered assemblies attractive as insulating ion-transport media in memristors, which operate based on the reversible electrochemical formation or annihilation of conductive filaments bridging between the active (top) and inert (bottom) electrodes under electric inputs to dynamically modulate the resistance across the device. DFT simulations indicate that the energy barrier for ions to diffuse through an octagonal carbon ring is drastically lower than crossing the benzene rings (0.3 eV versus 14.6 eV, see Supplementary Fig. 14 and Methods). Therefore, these large-size carbon rings, rich in both single and bilayer regions of ultrathin quasi-2D amorphous carbon nanomembranes, can provide atomic-scale predefined and continuous 1D channels for the filament formation, whose density can be drastically tuned by simply varying the number of layers stacked together, to reduce the device switching spatiotemporal variability (Fig. 6a)^35^. We fabricated arrays of 100 × 100 nm^2^ and 200 × 200 nm^2^ size memristors with merely two layers of sequentially deposited ultrathin quasi-2D amorphous carbon (total thickness 0.8–1.2 nm) conformally sandwiched between the Pt bottom electrode and the Ag top electrode on a 3-in. wafer (Fig. 6b, c, Methods). They exhibited uniform and forming-free bipolar resistive switching under low set/reset voltages below 0.4 V (Fig. 6d, e, note that dielectric breakdown instead of resistive switching was observed in films thicker than three atomic layers instead which are more suitable to function as robust gate dielectrics as illustrated in Figs. 4 and 5). In particular, the overall distributions of switching voltages had a small standard deviation less than 40 mV (Fig. 6f), which is superior to memristors based on either bulk 3D insulators or polycrystalline h-BN^36–38^. In addition to the small cycle-to-cycle variability, high spatial uniformity was also achieved. The averaged set/reset voltages were mapped for a 4 by 8 array (Fig. 7a). The standard deviations for device-to-device variability were less than 50 mV (Fig. 7b). All the functional devices (yield = 75%) exhibited small cycle-to-cycle variability with the average standard deviation of 50 mV for set (Fig. 7c) and 25 mV for reset (Fig. 7d). In addition, they showed a narrow distribution of on- (Fig. 7e) and off-state (Fig. 7f) resistance, with a large contrast above 200 times even in the worst-case scenario considering both device-to-device and cycle-to-cycle variabilities (Fig. 7g). The failed devices were largely caused by the delamination of the deposited Ag electrode from the dangling-bond-free amorphous carbon surface during the metal lift-off step (Fig. 7h).

**Fig. 8:**
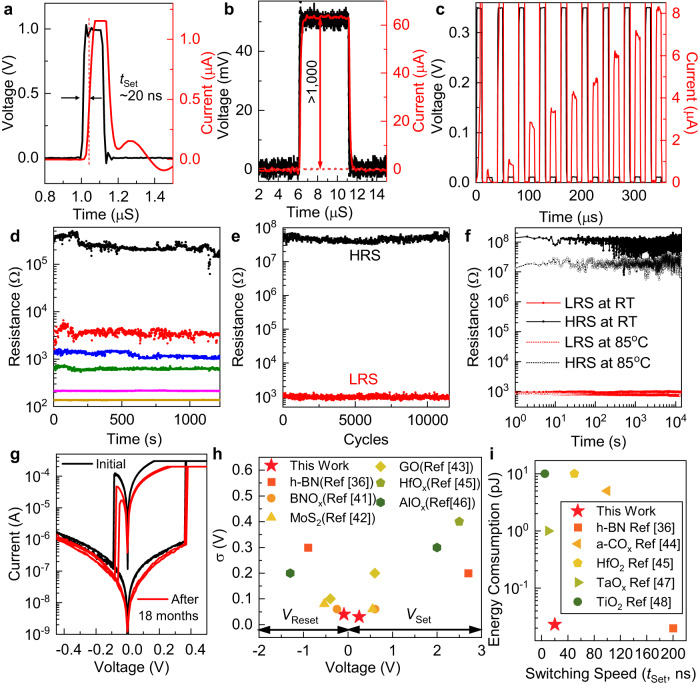
Speed, multilevel switching, rendition, endurance, and benchmark of memristors employing quasi-2D amorphous carbon bilayers as the ion-transport media. **a** Switching of the memristor with a 100 ns/1 V voltage set pulse to extract the set time (*t*_set_). The voltage pulse is shown in black in the left axis and the response current is shown in red in the right axis. **b** Read current (red, right axis) contrast of the memristor under the 10 µs/50 mV read voltage pulse (black, left axis) before (dashed lines) and after (solid lines) the set transaction shown in (**a**). **c** The multilevel switching of a Pt/quasi-2D amorphous carbon bilayer/Ag memristor with a sequence of 10 µs/0.35 V set pulses followed by 10 µs/50 mV read pulses. The voltage pulse is shown in black in the left axis and the response current is shown in red in the right axis. **d** Retention of these intermediate conductance states measured at room temperature. Different colors represent different resistance levels. **e** Endurance test showing >10^4^ cycles of stable operations between the HRS (black) and LRS (red) in ambient. **f** Change of the resistance of HRS (black) and LRS (red) as a function of time, measured at room temperature (RT, solid) and 85 ^o^C (hollow), respectively. **g** Memristive switching characteristics of two devices after 18 months storage (red) as compared with what was obtained right after completing the device fabrication (black). **h** Benchmarking operational set (*V*_Set_) and reset (*V*_Reset_) voltages and the associated standard deviations (*σ*) of memristors built on various 2D/3D materials. **i** The switching speed in terms of set time (*t*_Set_) and the associated energy consumption to perform the write operation to these memristors.

In addition to reducing the switching voltages and their variability, the intrinsic ultrathinness of quasi-2D amorphous carbon bilayer and the existence of predefined vertical filament pathways also improve the device operating speed and reduce the energy consumption, by limiting the number of atoms involved and their required drifting distance to modulate the filament formation^39^. For 100 ns-wide voltage pulses with 1 V amplitude under a current compliance of 1 µA, the set time was ~20 ns and it is mainly limited by the parasitic capacitance rather than the intrinsic switching speed of the device (Fig. 8a and Supplementary Fig. 15). The energy consumption per set transaction is therefore less than 20 fJ, and it is sufficient to induce a resistive switching with contrast more than 10^3^ times (Fig. 8b). The intermediate resistive states were accessible with the application of voltage pulses with smaller amplitude of 0.35 V without reset (Fig. 8c), and they have stable data retention for energy-efficient neuromorphic computing (Fig. 8d, the fluctuation in HRS is caused by noise during measurement)^40^. These attractive switching characteristics were obtained without sacrificing device stability. The memristors based on nanometre-thick quasi-2D amorphous carbon bilayer can be continuously switched between on- and off-states for more than 10^4^ cycles (Fig. 8e, better than memristors built on h-BN and comparable to flash memory), with good retention (>10^4^ s, no sign of degradation toward longer time) at both room temperature and 85 ^o^C (Fig. 8f), and no degradation was observed after storing the device in nitrogen box for 18 months (Fig. 8g). The simultaneous realization of all these memristor-performance metrics, including the high spatiotemporal uniformity under low operating voltage (Fig. 8h), fast speed together with low energy consumption (Fig. 8i), good endurance, and stable data retention has not been accomplished with devices built on other 2D materials (h-BN, MoS_2_, graphene oxide)^36,41–43^ or 3D bulk oxides (oxygenated amorphous carbon, HfO_2_, Al_2_O_3_, TaO_2_, TiO_2_)^44–48^ (See also supplementary Note 10 and Supplementary Table 4).

## Conclusion

In summary, we reported a strategy for preparing ultrathin quasi-2D amorphous carbon with thickness down to 1–2 atomic layers and their few-layered assemblies with precisely controlled film thickness on wafer scale. Compared to the vacuum-based growth approaches, this solution-based deposition scheme, despite forming monolayered films with a mixture of one and two atomic-layer thick regions, offers substantial advantages in terms of wafer-size scalability, low cost, and especially the capability to form multilayers with precisely controlled thickness in a layer-by-layer fashion, which are critically important for their technological applications. Despite the presence of some local single-atomic-layer level thickness fluctuations, their intrinsic ultrathinness, amorphous atomic structures, low density of surface dangling bonds, and exceptional physical properties make solution-prepared quasi-2D amorphous carbon attractive dielectrics for nanoelectronic devices including 2D-material-based transistors and memristors. In these proof-of-concept use-case demonstrations, compared to amorphous bulk metal oxides, the ultrathin amorphous carbon films with predominant sp^2^ carbon content can form clean interfaces with graphene and 2D metal chalcogenides, which leads to enhanced carrier transport mobility and minimum hysteresis. Compared to polycrystalline 2D h-BN, they have better processability and their amorphous atomic structures lead to drastically lower leakage current density at the same thickness with higher dielectric strength for substantially reduced power consumption and improved device reliability. Meanwhile, their intrinsic ultrathinness and distinctive atomic structures composed of heterogeneous carbon rings offer predefined filament formation pathways across atomically thin bilayer films, leading to drastically enhanced switching uniformity, reduced energy consumption, and faster operating speed as the switching media for electrochemical memristors. In addition to functioning as versatile solution-deposited nanodielectrics for electronics, these continuous atomically thin amorphous carbon nanomembranes would also be attractive for applications such as catalyst support and water purification with their ultra-strong mechanical properties and tuneable heterogeneous atomic structures^49,50^.

## Methods

### Preparation of carbon dots from coal char as solution-processable precursors

**Fig. 9:**
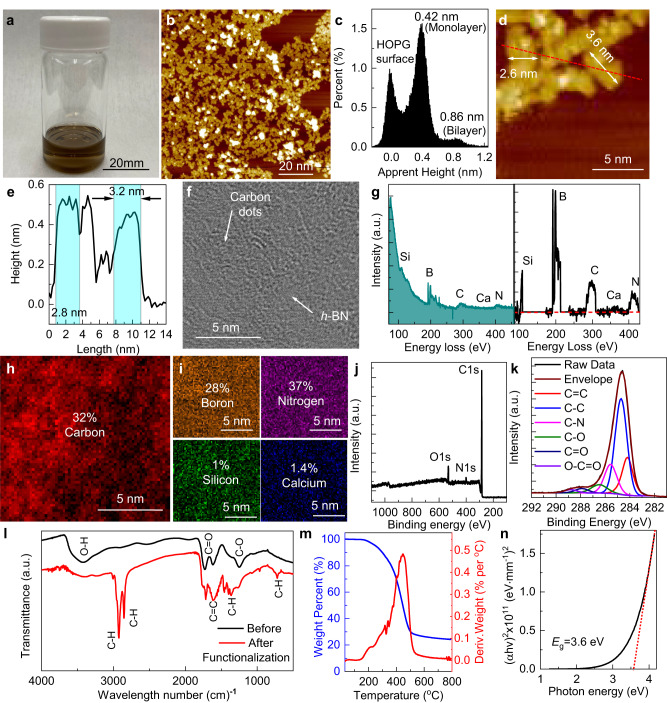
Coal-derived carbon-dot precursors. **a** Optical image of the carbon dot dispersion in toluene with a concentration of 0.05 mg mL^−1^. **b** STM image of carbon dots deposited on a HOPG substrate by spin casting. **c** Histogram of the measured apparent height, with zero referenced to the HOPG surface. Zoom-in view showing individual particles (**d**) and the associated linecut height profile (**e**) along the red dashed line. The blue-shaded areas highlight the width of two dots. STEM image (**f**), associated EELS spectrum (spectrum before and after background subtraction is displayed in the left and right frame, respectively), and the spatial mapping of carbon (**h**) and other elements identified (**i**) of carbon dots deposited on a suspended h-BN membrane to verify the purity. **j** XPS survey spectrum of carbon dots after functionalization. **k** Deconvoluted C1*s* peak (black) showing the contributions from sp^2^ and sp^3^ carbon as well as those having covalent bonds with O and N. **l** FTIR spectra of carbon dots before (black) and after (red) functionalization with oleylamine. **m** TGA of the functionalized carbon dots showing both the relative weight loss (blue, left axis) and its derivative (red, right axis). **n** Tauc plot with extrapolation (red dashed line) to determine the optical bandgap of coal-derived carbon-dot precursors.

Coal-derived carbon dots were synthesized by chemical oxidation of Blue Gem coal char (Carbon Technology Company, derived from bituminous Blue Gem coal mined from south-eastern Kentucky, U.S., which is a speciality coal with low, i.e., <1.5%, mineral content) using a mixture of concentrated sulfuric acid and nitric acid^10^. The process starts from grinding Blue Gem coal char into fine powders with size less than 100 mesh. Five grams of ground coal char was then added into 200 mL of concentrated H_2_SO_4_ (95–98%) and HNO_3_ (70%) obtained from Sigma-Aldrich (3:1 volume ratio) under mixing, and the mixture was heated to 100 °C in an oil bath for 24 h. Afterwards, the mixture was naturally cooled to room temperature and then diluted by 20 times with deionized (DI) water. This diluted mixture was subsequently centrifuged at 5000 r.p.m. for 10 min to remove unreacted carbon materials and insoluble minerals. The supernatant was collected and neutralized by NaOH to a pH of 2. The obtained dispersion was then purified using a tangential crossflow ultrafiltration system (Spectrum® KR2i/ KMPi TFF Systems, Repligen) with a 1 kD filtration membrane. This continuous-flow ultrafiltration process allows for very efficient and rapid removal of the soluble metal-ions and small-molecule organic impurity contents^51,52^. After three rounds of ultrafiltration, the pH of the dispersion was adjusted with 1 mol L^−1^ HCl to a pH of 2, followed by five additional ultrafiltration rounds to obtain carbon-dot precursors with high level of purity. The yield of the finally obtained coal-derived carbon dots in aqueous dispersion was approximately 10 wt%. To functionalize these as-synthesized carbon dots and disperse them in organic solvents, oleylamine (Sigma Aldrich) dissolved in 5 mL of ethanol was added into a 50 mL aqueous dispersion of carbon dots (concentration = 2 mg mL^−1^) under vigorous mixing at an oleylamine to carbon dot ratio of 2:1 by weight. The mixture was then heated to 80 °C for 2 h. After cooling to room temperature, the mixture was vacuum-filtered. The collected filter cake was heated at 130 °C in air for 30 min to complete the amidation reaction, and then redispersed into a mixture of toluene and ethanol (toluene to ethanol volume ratio of 20–40:1) at a concentration of 4 mg mL^−1^ to form a stable dispersion of functionalized carbon dots, which can be further diluted for subsequent depositions (Fig. 9a). Transferring the functionalized carbon dots from aqueous to toluene further reduced the metal-ion impurity content, which is typically not soluble in organic solvents. The diameter of the prepared carbon dots is in the range from 2–4 nm, and the predominant of them is one atomic layer thick, as evident from the STM micrographs showing individual dots deposited by spin casting from a low concentration dispersion on a freshly peeled highly oriented pyrolytic graphite (HOPG) substrate (Fig. 9b–e). The carbon-dot precursors were also deposited using the same spin-coating process on a h-BN support transferred on a holey-carbon TEM grid for STEM imaging (Fig. 9f). Although it is difficult to resolve individual particles due to their poor radiation stability even under a low operational voltage of 60 kV, the EELS mapping confirms the high purity of the carbon-dot precursors, with silicon and calcium as the only metal-element impurities with abundance above the EELS detection limit (Fig. 9g–i and Supplementary Fig. 16). The XPS survey spectrum also shows predominant C1*s* peak, together with minor O1*s* and N1*s* peaks reflecting the presence of amide bonds connecting the carbon dots with oleylamine surfactants (Fig. 9j). The elemental composition is 88% carbon, 8.6% oxygen, and 3.4 % nitrogen. The deconvoluted high-resolution C1*s* peak displays not only sp^2^ carbon (C=C), but also sp^3^ aliphatic (C–C) and nitrogenated carbon (C–N) together with a small amount of oxygenated carbon (C–O, C=O, and O–C=O), which are likely correlated with the oleylamine functionalization and will disappear after the coalescing process (Figs. 9k and 1c). Figure 9l compares the Fourier-transform infrared spectroscopy (FTIR) spectra of the coal-derived carbon dots before and after oleylamine functionalization. The substantial reduction of the O–H, C=O, and C–O peak intensities, together with the appearance of prominent C–H peaks, indicates that most of the carboxyl groups around the edges of the carbon dots have been converted to amide bonds connecting with the long alkyl chain of oleylamine^53^. These amide bonds are thermally unstable and will be scissored at ~450–500 ^o^C (Fig. 9m), which is the temperature required to coalesce the functionalized carbon dots together to form the continuous ultrathin quasi-2D amorphous carbon. The optical transmittance of the prepared coal-derived carbon dots was measured by drop casting a thick film on a sapphire substrate. The extracted effective optical bandgap is 3.6 eV, reflecting the quantum confinement effect (Fig. 9n).

### The formation of ultrathin quasi-2D amorphous carbon with thickness down to 1–2 atomic layers and their few-layered assemblies from solution-processable carbon-dot precursors

**Fig. 10:**
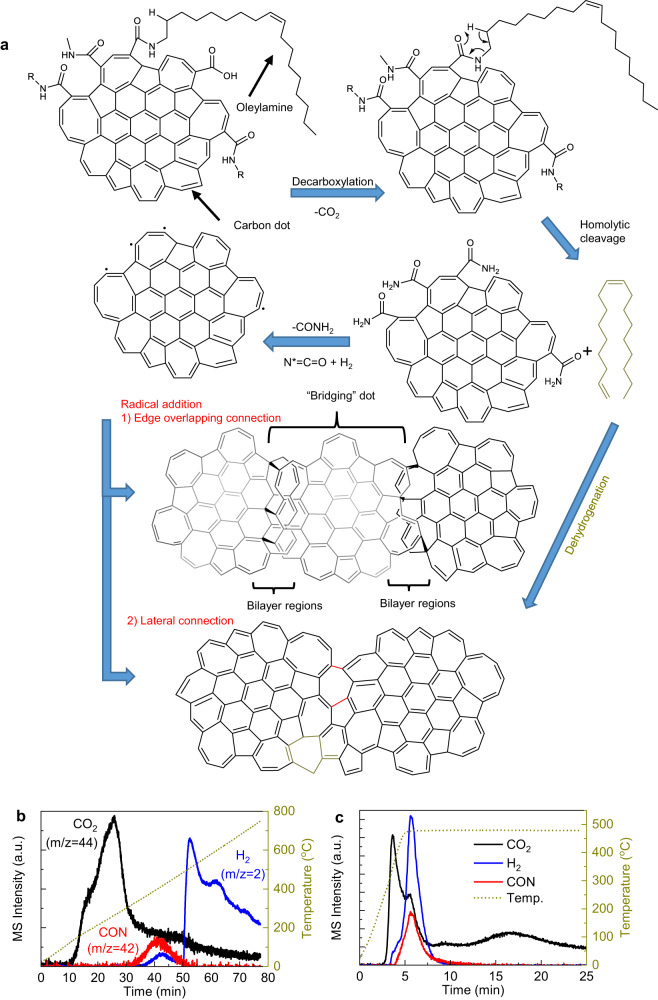
Coalescence of carbon dots upon annealing forms an atomically thin quasi-2D amorphous carbon film. **a** Schematic illustrating the proposed thermal decomposition and crosslinking mechanism. TGA-MS spectrograms of the functionalized carbon dots obtained with temperature either slowly increased from room temperature to 800 °C with a low ramping rate of 10 ^o^C min^−1^ (**b**) or rapidly increased to 500 °C with a fast-ramping rate of 100 °C min^−1^ followed by holding at 500 °C for 20 min to mimic the film preparation conditions (**c**). MS intensities of the dominant volatile decomposition products are assigned to CO_2_ (black), CON* (red), and H_2_ (blue) based on their different mass-to-charge (*m*/*z*) ratios.

A silicon wafer covered with 90 nm thermal oxide was cleaned with piranha solution and then functionalized with HMDS using the Yield Engineering vapor prime oven. The carbon-dot precursor solution with a concentration of 0.05 mg mL^−1^, which was selected to form a single layer with thickness down to 1–2 atoms (see Supplementary Note 11 and Supplementary Fig. 17 for more detailed discussion), was dispersed on the substrate and spin-coated at 3000 r.p.m. for 60 s in cleanroom. After drying, the film was then soft-baked at 120 °C for 15 min to get rid of the solvent residue, and finally annealed under 500 °C within a nitrogen-filled glove box (oxygen and water levels were both below 1 p.p.m.) for 10 min to coalesce the deposited carbon dots into a macroscopically uniform and continuous quasi-2D film. The reaction mechanism is illustrated in Fig. 10a. Upon annealing, the process starts from decarboxylation, followed by the homolytic scission of the amide bond through the *cis*-elimination, where a weakened NH–CH_2_ bond in the *β* position with respect to the carbonyl group is cleaved through hydrogen transfer assisted by a six-membered ring transition state. Afterwards, the cleavage of the C–CO amide bond then leads to the evolution of a CONH_2_ fragment and the formation of radicals, as in the pyrolytic decomposition of nylon^54^. The radicals can subsequently attack the neighboring carbon dots with either overlapping (most likely) or contacting edges and unsaturated bonds to cross-link them together through radical addition, forming the continuous quasi-2D amorphous carbon film with thickness therefore fluctuating between single and double atomic layers^16,55^. Meanwhile, the released aliphatic hydrocarbon chains are pyrolyzed into graphitic carbon^56^, which further serves as carbon feedstock to seal the geometric gaps between as-deposited carbon dots as predicted by molecular dynamics simulation^16^. This mechanism is supported by examining the volatile reaction products utilizing Thermogravimetric Analysis (TGA) coupled with Mass Spectrometry (MS), as shown in Fig. 10b. Carbon dioxide evolved starting from 200–400 ^o^C, resulting from the decomposition of the residue carboxyl groups. At ~400 ^o^C, the concurrent evolution of 42 and 2 mass units was observed, which is associated with the further decomposition of the CONH_2_ fragments into isocyanato radicals (N*=C=O) and hydrogen^54^. At 500 ^o^C, the sudden increase of the hydrogen evolution can be correlated with the pyrolysis of the released hydrocarbon ligands into amorphous graphitic carbon^56^. Under a heating profile mimicking the experimental conditions in film synthesis, the evolution of all these volatile species was observed and the pyrolysis-crosslinking process was largely completed within 10 min (Fig. 10c). The mechanism also explains the XPS results suggesting that the carbon dot assembly, which contains both sp^2^ and sp^3^ carbon, is converted to a film predominantly composed of sp^2^ carbon. Multilayered quasi-2D amorphous carbon films were constructed by repeating the spin casting and annealing process after forming the first layer as described above. Single-crystalline *c*-plane sapphire wafers (2-in. diameter, Universitywafer) were used as substrate to prepare samples for cross-sectional TEM/STEM, which give a flatter and clearer interface with the deposited ultrathin quasi-2D amorphous carbon film compared to amorphous SiO_2_ on silicon.

### Transfer of ultrathin quasi-2D amorphous carbon with thickness down to 1–2 atomic layers and few-layered thin films

A thin film of methyl methacrylate (MMA, Kayaku Advanced Materials) was spin casted on top of the quasi-2D amorphous carbon formed on a blanket SiO_2_/Si wafer at 2000 r.p.m. and then baked under 180 °C for 3 min. By selectively etching the support SiO_2_ in concentrated HF (49%), the MMA/quasi-2D amorphous carbon stack was lifted off from the wafer surface as a freestanding floating membrane. After washing with DI water, the hybrid film was picked up by the target substrate, e.g., holey-carbon grid for TEM (Supplementary Fig. 7) or the perforated silicon for nanoindentation measurement (Fig. 3a). After drying the film in a desiccator, the MMA top layer was removed by decomposition in an atmospheric-pressure furnace flowing 100 s.c.c.m. H_2_ and 100 s.c.c.m. Ar simultaneously at 400 °C for 2 h. A low-temperature ramping rate of ~4 °C min^-1^ was adopted to minimize the thermal shock.

### TEM/STEM characterizations

The low-magnification ADF-STEM imaging of suspended ultrathin quasi-2D amorphous carbon with thickness down to 1–2 atomic layers on holey-carbon TEM grid (Quantifoil with 600 nm diameter circular holes) was conducted with FEI/TFS Titan Themis TEM/STEM. The operational voltage was 200 kV. The STEM cross-sectional micrographs of the atomically thin quasi-2D amorphous carbon and its multilayers on sapphire were recorded using the FEI Themis Z analytical TEM/STEM with a high brightness Schottky field-emission electron source operating at 300 kV in the STEM NanoProbe SA Zoom Diffraction Mode. The detector was the high-angle annular dark-field (HAADF) STEM detector with convergence angle of 18 mrad. The dwell time was 4 μs and the frame time was 20.1 s in total. The camera length was 115 mm. The cross-sectional TEM images of quasi-2D amorphous carbon on sapphire were obtained using the Hitachi H-9500 Dynamic Environmental TEM with the Orius SC200 CCD camera. The operational voltage was also 300 kV, while the exposure time for each image was 1 s for the single layer and 0.5 s for multilayers, respectively. The cross-sectional samples were prepared using the FEI Helios 600i dual-beam focused-ion beam (FIB). The high-resolution STEM images and EELS spectra of the suspended ultrathin quasi-2D amorphous carbon were collected using the Nion UltraSTEM 100 electron microscope operated at 60 kV and equipped with field-emission gun, 5th order aberration corrector, and a Gatan Enfina EELS spectrometer. The low operation voltage of 60 kV and the ultrahigh vacuum chamber of Nion microscope minimized the radiation damage to carbon film during imaging^57–59^. Data were acquired simultaneously using both a bright-field detector with collection angles 0–7 mrad and a HAADF detector with collection angles 86–200 mrad. The images were originally recorded as 20-frame stacks. Bright-field image stacks were then registered using cross-correlation and averaged. Due to presence of a few surface impurity atoms still mobile at 60 kV, it was not possible to directly cross-correlate HAADF images. However, using the shifts computed from cross-correlating simultaneously acquired bright-field images to register and then average dark-field stacks, similar improvement in signal-to-noise parameters was achieved. The aligned stacks were combined and then filtered to improve the contrast of the carbon rings using a custom linear filter with a central peak that matches approximately the STEM image resolution and is surrounded with a ring of negative intensities for contrast sharpening. EELS spectra were analyzed using the Gatan software suite. The STEM images and EELS spectra of carbon-dot precursors were also taken by this equipment at 60 kV. Here a crystalline h-BN membrane was firstly transferred on a holey-carbon TEM grid, and the carbon dots were then deposited on top of the h-BN support by spin casting.

### Computational method

The unit cells were generated using the Visualization for Electronic and Structural Analysis program^60^, with the atomic coordinates provided in Supplementary Data 1. First principles calculations were then performed using DFT as implemented in the Vienna Ab Initio simulation package (VASP)^61,62^. Exchange and correlation were treated using the generalized-gradient approximation parametrized by Perdew–Burke–Ernzerhof^63^, and the projector-augmented-wave method was used to model the electron-ion interaction^64^. Convergence tests were performed for the cut-off energy of the plane-wave-basis, **k**-point grid, and vacuum size of the cell. Specifically, a 520 eV plane-wave cut-off energy, a 1 × 1 × 1 Γ centered **k**-point grid, and 10 Å vacuum in the direction perpendicular to the film were used in the calculation. The diffusion of a silver ion through a six-membered and eight-membered carbon ring embedded in the graphene matrix was modeled with the climbing-image nudged-elastic band method implemented in the transition-state-theory tools for VASP^65^. Cells with a single silver ion roughly 4.5 Å above and below the carbon layer represented the initial and final state of the diffusion pathway. Based on the conjugate-gradient algorithm, they were relaxed to a maximum force of 15 meV Å^−1^ (Supplementary Fig. 14a–f). With the movement of the silver ions restricted in the *z* direction, force-based optimizers were then used to find the minimum energy pathway with at least nine intermediate steps to generate the reaction coordinate plots (Supplementary Fig. 14g, h), where the energy barrier blocking the diffusion can be determined.

### Fabrication of graphene transistors with either SiO_2_ bottom-gate dielectric or quasi-2D amorphous carbon top-gate dielectric

Graphene monolayer (Grolltex) grown on copper foil by CVD was transferred to a heavily *p*-doped silicon wafer with 90 nm thermally grown SiO_2_ and prepatterned alignment marks. The transfer process started from etching the Cu foil in 0.2 mol L^−1^ ammonium persulfate (NH_4_)_2_S_2_O_8_ solution, with a polymethyl methacrylate (PMMA, Kayaku Advanced Materials A3 950k, 3 wt% in anisole) film spin casted on top as the mechanical support. After washing with DI water, the graphene/PMMA film was picked up from the water bath using the Si/SiO_2_ target substrate, air-dried in a chemical hood, and then baked under 80 °C for 30 min to improve the adhesion. The PMMA overlayer was removed by acetone, followed by annealing in hydrogen furnace under 400 °C for 2 h to remove the organic residues. A photolithography step was then performed to pattern the photoresist (AZ 5214E) into etching masks covering the channel region of each device. A subsequent oxygen plasma etching removed graphene in the unprotected areas for device isolation, and the photoresist was stripped by soaking the substrate in acetone. Another photolithography step was performed to define the source-drain electrode patterns into the photoresist, and an electron-beam evaporation (Temescal) was used to deposit 0.2 nm Ti/20 nm Pd/10 nm Au, followed by lift-off in acetone to form the source-drain contacts to the graphene channel. It completed the fabrication of bottom-gated graphene transistors. For top-gated graphene transistors and graphene/quasi-2D amorphous carbon film/metal capacitors, multiple layers of quasi-2D amorphous carbon were consecutively deposited on top in a layer-by-layer fashion to the desired thickness. A final lithography step was performed to define the metal gate electrode sitting on top of the quasi-2D amorphous carbon dielectric again by lift-off.

### Fabrication of MoS_2_ transistors with Si_3_N_4_ bottom-gate dielectric and quasi-2D amorphous carbon top-gate dielectric

High-quality monolayer MoS_2_ was grown on sapphire by CVD^66^. The MoO_3_ powders (99%, Aldrich) were placed in a quartz boat located in the heating zone center of the furnace. The sulfur powders (99.5%, Alfa) were placed in a separate quartz boat at the upper stream side of the furnace and the temperature was maintained at 140 °C during the reaction. The sapphire substrates for growth were put on the downstream side, next to the quartz boat containing MoO_3_. The precursor was carried by Ar flowing gas (90 s.c.c.m.), and the chamber pressure was kept at 30 Torr. The center heating zone was heated to 740 °C. After reaching growth temperature, the heating zone was kept for 5 min and the furnace was then naturally cooled down to room temperature. The as-synthesized MoS_2_ monolayer was transferred to a heavily *p*-doped silicon wafer covered with 100 nm Si_3_N_4_ deposited by low-pressure CVD. The transfer process started from spin-casting a PMMA film on top of the MoS_2_ as the mechanical support. The PMMA/MoS_2_ bilayer was subsequently released from sapphire substrate by etching in ammonia aqueous solution at 80 °C, and picked up by the receiving substrate. An annealing at 70 °C for 30 min was performed to remove moisture and improve the adhesion between the MoS_2_ and Si_3_N_4_ post-transfer. The PMMA overlayer was removed by dissolving in hot (60 °C) acetone. A photolithography step was then performed to define the photoresist (AZ 5214E) as the source-drain electrode patterns. 10 nm Ni contact with 10 nm Au capping layer was deposited by electron-beam evaporation at high vacuum (~1 × 10^−^^7^ torr) followed by lift-off in hot acetone. A second photolithography step was performed to pattern the photoresist into etching masks covering the channel region of each device. A subsequent oxygen plasma (70 W, Zepto M2, Diener Electronics) removed MoS_2_ in the unprotected area for device isolation, and the photoresist was stripped by soaking the substrate in acetone. Five layers of atomically thin quasi-2D amorphous carbon formed on another SiO_2_/Si substrate were then transferred on top with the MMA as the support. The transfer of the quasi-2D amorphous carbon multilayer was repeated once to get the total top-gate dielectric thickness to 4–5 nm. A final lithography step was performed to define the aluminum gate electrode sitting on top of the quasi-2D amorphous carbon dielectric again by lift-off.

### Fabrication of memristors employing quasi-2D amorphous carbon bilayer as the ion-transport media

An electron-beam lithography (EBL) step was first performed to define the bottom electrode pattern into a bilayer resist of polydimethylglutarimide SF6 and PMMA 950k A4 (Kayaku Advanced Materials), and electron-beam evaporation (Lesker PRO Line PVD 75) was used to blanketly deposit 1 nm Ti and 20 nm Pt, followed by lift-off in acetone. The large contact pads to these bottom electrodes were then defined by photolithography and metal lift-off. After completing the fabrication of the bottom Pt electrodes, an oxygen plasma RIE was performed to clean the surface, followed by the HMDS functionalization. A quasi-2D amorphous carbon bilayer (thickness 0.8–1.2 nm) was then deposited on top by repeating the layer-by-layer spin-casting-coalescence process twice. Since the quasi-2D amorphous carbon film was formed directly on the Pt bottom electrodes, it avoids the transfer process which could lead to the formation of macroscopic defects and thus limit the device yield. Afterwards, another EBL step was performed to define the top electrodes, together with the lift-off of the electron-beam evaporated Ag (Temescal). 150 nm SiO_2_ was then deposited again by electron-beam evaporation (AJA ATC-E) as a device passivation layer. A final photolithography step created 100 µm by 100 µm windows into the photoresist (AZ5214), followed by wet etching of evaporated SiO_2_ by buffered oxide etchant to open the probing pads for device measurements.

### Instrumentation

Raman spectra were taken using a Nanophoton 11 micro-Raman under excitation of a 532 nm laser with a spot size of about 1 × 1 μm^2^. AFM topography images of quasi-2D amorphous carbon were measured in tapping mode using an Asylum Research Cypher, with S-Tap300Al tapping tips from BudgetSensors. The nanoindentation was performed using the same equipment but in the contact mode with D-tips (spring constant of 0.03–0.12 N m^−1^, nominal tip radius of 2 nm, frequency of 12–24 kHz) of the Sharp Nitride Lever (SNL-10) probes from Bruker. AFM images of MoS_2_ were acquired using a Nanosurf Easyscan AFM in the tapping mode using tips with a spring constant of 0.4 N m^−1^. Raman and photoluminescence spectra of MoS_2_ were collected with a laser wavelength of 488 nm and 300 grooves per mm grating in a WITec apyron Confocal Raman Microscope. XPS spectra of the ultrathin quasi-2D amorphous carbon formed on Si/SiO_2_ substrate were measured using a Kratos Axis ULTRA spectrometer with hybrid spherical capacitor electron energy analyzer. The sp^2^ carbon peak position was internally calibrated using the XPS peak measured from a crystalline graphene monolayer transferred onto the same SiO_2_/Si substrate. XPS spectra of the coal-derived carbon-dot precursors were obtained with a PHI 5600ci spectrometer equipped with a hemispherical electron analyzer and a monochromatic Al Kα (1486.6 eV) radiation source. The pass energy of the analyzer was 55 eV. XPS spectra of MoS_2_ were acquired using a Phi 5400 spectrometer. The energy scale of the XPS spectra was calibrated by setting the 4*f*_7/2_ line of clean gold and the 2*p*_3/2_ line of clean copper located at 84 and 932.67 eV, respectively. SEM micrographs were acquired using a Hitachi S4800 microscope. Direct-current measurements, capacitance–voltage measurements, and voltage-pulse measurements were all performed in ambient using a manual probe station (LakeShore Cryotronics CRX–6.5 K) connected with a semiconductor parameter analyzer (Keysight B1500A) equipped with integrated high-resolution source-measurement units (Keysight B1517A), multi-frequency capacitance-measurement unit (Keysight B1520A), and waveform generator/fast-measurement unit (WGFMU, Keysight B1530A). The WGFMU module has a built-in current-to-voltage sampling circuit that allows it to directly measure the current flowing across the memristor with a sampling interval of 5 ns and resolution of 14 nA, while simultaneously applying the square voltage waveform. Optical properties of the ultrathin quasi-2D amorphous carbon and the carbon-dot precursors were recorded using either ellipsometry (J.A. Woollam VASE) or ultraviolet-visible absorbance spectrophotometer (Agilent Cary 5000). STM was performed using an integrated surface analysis tool built by Omicron Nanotechnology GmbH. FTIR was carried out using a KBr medium on a Cary 670 FTIR spectrometer from Agilent Technologies. TGA was conducted through a Q50 Thermogravimetric Analysis System with a temperature range up to 800 °C in a N_2_ atmosphere. TGA-MS results were obtained with a Linseis TGA HS 1600 coupled with a Pfeiffer Vacuum OmniStar MS. The measurement was carried out within an Ar environment with the continuous flow of ultrapure Ar at 100 cc min^−1^. PMMA photoresist was patterned with a Raith e-line EBL system and an Elionix ELS-G150 150 keV EBL system. The AZ 5214E photoresist was patterned with a Heidelberg MLA150 aligner. The DFT simulations were performed on the Illinois Campus Cluster.

### Supplementary information

Supplementary Information

Description of Additional Supplementary Files

Supplementary Data 1

## Data Availability

The data that support the findings of this study are available from the corresponding author upon reasonable request. The atomic coordinates of models used in electronic structure calculations and molecular dynamic simulations are provided in Supplementary Data 1.
