# Supplementary material for: Ultrathin quasi-2D amorphous carbon dielectric prepared from solution precursor for nanoelectronics
Source: Commun Eng. 2023 Dec 20;2:93. doi: 10.1038/s44172-023-00141-9 (PMC10955813; doi:10.1038/s44172-023-00141-9)
Supplement: Supplementary file 1 — Supplementary Information [file 44172_2023_141_MOESM1_ESM.pdf]

Ultrathin Quasi-2D Amorphous Carbon Dielectric Prepared from Solution Precursor for Nanoelectronics (Online Supplementary Information)

Fufei An[1], Congjun Wang[2], Viet Hung Pham[2,3], Albina Borisevich[4], Jiangchao Qian[1], Kaijun Yin[1], Saran Pidaparthy[1], Brian Robinson[1], Ang-Sheng Chou[5], Junseok Lee[2,3], Jennifer Weidman[2,3], Sittichai Natesakhawat[2,3], Han Wang[5], André Schleife[1,6,7], Jian-Min Zuo[1,6], Christopher Matranga[2], and Qing Cao [1,6,8,9,10*]

[1]Department of Materials Science and Engineering, University of Illinois Urbana-Champaign, Urbana, IL, USA. [2]National Energy Technology Laboratory (NETL), Pittsburgh, PA, USA. [3]NETL Support Contractor, Pittsburgh, PA, USA. [4]Center for Nanophase Materials Sciences, Oak Ridge National Laboratory, Oak Ridge, TN, USA. [5]Corporate Research, Taiwan Semiconductor Manufacturing Company (TSMC), Hsinchu, Taiwan. [6]Frederick Seitz Materials Research Laboratory, University of Illinois Urbana-Champaign, Urbana, IL, USA. [7]National Center for Supercomputing Applications, University of Illinois Urbana-Champaign, Urbana, IL, USA. [8]Department of Electrical and Computer Engineering, University of Illinois Urbana-Champaign, Urbana, IL, USA. [9]Department of Chemistry, University of Illinois Urbana-Champaign, Urbana, IL, USA. [10]Holonyak Micro & Nanotechnology Laboratory, University of Illinois Urbana-Champaign, Urbana, IL, USA.

**e-mail: qingcao2@illinois.edu*

Supplementary Note 1: Raman mapping of quasi-2D amorphous carbon.

Raman spectroscopy has been demonstrated as a reliable approach to characterize the thickness of few-layer graphene with high level of precision and high throughput[1], and it is also applicable toward the characterization of quasi-2D amorphous carbon. As shown in Supplementary Fig. 1a, the intensity of the *D* and *G* peaks of the prepared quasi-2D amorphous carbon films increases superlinearly with the increase of the number of layers deposited on SiO_2/Si substrate, normalized to the intensity of the peak corresponding to the second-order

Raman scattering by optical phonons of the silicon substrate. Quantitatively, the ratio of integrated intensity of the Raman peaks of quasi-2D amorphous carbon films and the silicon peak has a clear correlation with both the film thickness determined by AFM and the number of layers controlled by the layer-by-layer deposition process (Supplementary Fig. 1b). Based on this correlation, we can convert the distribution of the Raman intensity across a 3-inch diameter wafer covered with an ultrathin quasi-2D amorphous carbon (Fig. 1a) into an approximate thickness distribution with standard deviation of 0.2 Å (Supplementary Fig. 1c). In addition to the homogenous integrated peak intensity, the ultrathin quasi-2D amorphous carbon prepared by our approach exhibits uniform I_D/I_G ratio on wafer scale (Supplementary Fig. 1d), with a normalized standard deviation less than 8% (Supplementary Fig. 1e), showing that the amorphous structure is also spatially uniform. The I_D/I_G ratio has a much less pronounced correlation with the film thickness, with the slight increase likely caused by the interference effect (Supplementary Fig. 1f)[2].

Supplementary Note 2: Cross-sectional STEM imaging of ultrathin quasi-2D amorphous carbon with thickness down to 1–2 atomic layers.

We used cross-sectional STEM to precisely determine the thickness of ultrathin quasi-2D amorphous carbon. We chose single-crystalline sapphire as the substrate to obtain a clear interface with the amorphous carbon film deposited on top. After forming the ultrathin quasi-2D amorphous carbon following the process described in Methods, a layer of polycrystalline platinum was deposited by sputtering to protect the ultrathin quasi-2D amorphous carbon from the FIB-cutting process. The FIB-cutting direction is schematically illustrated in Supplementary Fig. 2a, as determined from the electron diffraction patterns. The expected crystal structure of the exposed sapphire substrate shows regular atomic planes of aluminium atoms with 2.2 Å pitch (Supplementary Fig. 2b), which agrees with what we observed in high-resolution STEM images (Supplementary Fig. 2c). Meanwhile, the atomic planes of the crystalline platinum film on top of the ultrathin quasi-2D amorphous carbon can also be visualized under the appropriate image brightness and contrast (Supplementary Fig. 2d). Since there is a large contrast difference, to

clearly show the atomic structures of both sapphire and platinum, we chose two contrast levels as in Supplementary Fig. 2c and 2d, and then combined them pixel by pixel to highlight the interface with the ultrathin quasi-2D amorphous carbon sandwiched in between (Fig. 1d), from which the ultrathin quasi-2D amorphous carbon film thickness can be precisely determined (Fig. 1e). In comparison, in the control sample where the Pt film was deposited directly on top of pristine sapphire wafer, no such interfacial layer was observed (Supplementary Fig. 3). Similar results were also obtained from phase-contrast TEM. Such clear difference with negative controls confirmed what we observed is indeed the deposited ultrathin quasi-2D amorphous carbon. The slightly blurring at the interface (thickness less than 0.1 nm) in the control samples is likely caused by the surface roughness of the sapphire substrate in the projection depth.

We obtained such high-resolution cross-sectional STEM images of ultrathin quasi-2D amorphous carbon at several different locations (Supplementary Fig. 4a-c). The film exhibited a uniform thickness at a scale much longer than the average size of the carbon-dot precursors (Supplementary Fig. 4d-f), suggesting that these carbon-dot precursors were laterally connected to from a uniform and continuous film, which could be assisted by some slightly overlapping regions along the edges of neighbouring carbon-dot precursors. The average film thickness was determined to be 4.1 Å, with a standard deviation of 0.4 Å (Supplementary Fig. 4g).

Supplementary Note 3: Pin-hole defect density in ultrathin quasi-2D amorphous carbon.

We used the wet-etching based chemical amplification to evaluate the area density of pin-hole type defects in the prepared ultrathin quasi-2D amorphous carbon[3]. An as-prepared ultrathin quasi-2D amorphous carbon with thickness down to 1–2 atomic layers was transferred to a SiO_2/Si substrate covered by a 200 nm thick film of Au deposited by electron-beam evaporation (Supplementary Fig. 6a), which was then subject to an etchant of Au (Transene gold etchant TFA). The chemical etching removed the Au film outside of the area covered by the transferred quasi-2D amorphous carbon (Supplementary Fig. 6b) and converted the pin holes in the carbon film into nearly micrometre-size defects in the Au film easily visible in optical microscopy (Supplementary Fig. 6c). There were also some macroscale cracks and wrinkles,

which were likely caused by the transfer process. The number of defects was quantified using the images taken by a 3D laser scanning confocal microscope (Keyence VK-X1000, Supplementary Fig. 6d). The average defect density measured was $(17\pm5)\times10^3$ per mm^2 (Supplementary Fig. 6e), which is fairly low for an atomically thin film and at least partially caused by the slight mechanical damage during the film transfer [4]. In comparison, it is only about 10 times higher than that of 2 nm thick Al_2O_3 deposited on Ni by ALD (1.2×10^3 defects per mm^2)[5]. Multilayers of quasi-2D amorphous carbon are required in device applications to ensure such pin-hole defects in each layer will not limit the device yield.

Supplementary Note 4: Electronic structures of idealized 2D amorphous carbon.

By examining the distribution of the ion-projected density of states (DOS), we find that although the 2D amorphous carbon model with embedded nanocrystallites (Supplementary Fig. 10a) demonstrates substantial DOS at energies close to the Fermi level (Supplementary Fig. 10b), most of these available states originate from states strongly confined in the amorphous regions lacking regular atomic structures, where a few atoms make dominant contributions (Supplementary Fig. 10c). The high degree of localisation of these available states diminishes the transmission probability for carriers, and it leads to weak coupling to the incident light radiation, due to small optical absorption cross-sections. In contrary, for deeper energy DOS (e.g., 4 eV below the Fermi level), a large portion of the atoms exhibit small projected DOS (Supplementary Fig. 10d), indicating that they form large-area delocalised states with large optical absorption cross-section, which leads to the optical bandgap observed in experiment (Fig. 3l).

Supplementary Note 5: Comparison of the physical properties of quasi-2D amorphous carbon and h-BN.

The physical properties, including interlayer spacings[6,7], Young's modulus[8,9], bandgap[10,11], out-of-plane dielectric constant[12,13], dielectric strength[14-19], and lateral resistivity[20,21], of quasi-2D amorphous carbon and h-BN are compared in Supplementary Table 1. They exhibit similar characteristics as both mechanically strong and electrically insulating, with intermediate

dielectric constant and bandgap. However, the quasi-2D amorphous carbon, with the intrinsic random atomic structures and the absence of sharp crystalline grain boundaries, enables drastically smaller leakage current and higher dielectric strength (Fig. 3h and 3i)[14-19]. The solution-based growth enables uniform wafer-scale deposition with precisely controlled film thickness (See Supplementary Note 1 and Supplementary Fig. 1). Such superior dielectric properties and scalability make quasi-2D amorphous carbon films potentially a more attractive candidate for applications as the ultrathin dielectric material in nanoelectronic devices (See Supplementary Note 7, Supplementary Note 9, and Supplementary Note 10 for more detailed discussions and benchmarks).

Supplementary Note 6: Verification of the effective field-effect mobility μ of graphene transistors extracted using the fitting method.

The mobility of graphene transistors has been typically extracted using a fitting method developed by Kim *et al.* in 2009, which has the capability to determine the parasitic contact resistance (R_c) and the residue carrier concentration (n_0, which also determines the quantum capacitance C_q) together with the effective field-effect mobility μ[22]. To verify the accuracy of the extracted mobility value by making sure it is not influenced by inaccurately determined R_c or n_0 in the fitting, we first used the transmission-line method to independently determine the device R_c and benchmark it with the fitting result. We fabricated bottom-gated and top-gated graphene transistors with different L_{ch} from 500 nm to 3.5 μm and identical W of 3.5 μm (Supplementary Fig. 11a), and groups of their device resistance (R_T)–gate overdrive (V_{GS}-V_{Dirac}) characteristics were then collected (Supplementary Fig. 11b and 11c). The total resistance R_T is modelled as the combination of the channel resistance and R_c. By linear fitting R_T of graphene transistors with different L_{ch} under the same gate overdrive, R_c can then be extracted from the y-axis intercepts (Supplementary Fig. 11d and 11e). The parasitic resistance for bottom-gated devices (ca. 500 Ω) was lower compared to their top-gated counterparts (ca. 1 k Ω). It is because the modulation of the graphene under the source-drain contacts by the gate electric field in the bottom-gate configuration helps to reduce R_c[23]. Quantitatively, the values of R_c extracted by

either the transmission-line or the fitting method agreed quantitatively well with each other (Supplementary Fig. 11f). We then independently determined n_0, which influences the extracted μ by affecting the graphene quantum capacitance C_q, from capacitance-voltage measurements[24]. We fabricated arrays of graphene/quasi-2D amorphous carbon film (five layers, total thickness ~ 2.4 nm)/metal capacitors (Supplementary Fig. 12a). Their major difference from the top-gated graphene transistors was the absence of any overlap between the top metal gate electrode and the metal contact pads to the graphene to minimize the parasitic capacitance resulting from fringing field, and the much larger device size to increase the overall capacitance for more accurate measurement. The gate leakage current density was still smaller than 10^{-4} A·cm^{-2}, even with more than 100 times larger device area (Supplementary Fig. 12a inset), showing the robustness of the multi-layered quasi-2D amorphous carbon films as dielectrics. The measured gate capacitance scaled linearly with the size of the overlapping area between the top metal electrode and graphene, indicating that the impact from the fringing field is indeed negligible (Supplementary Fig. 12b). Their capacitance-voltage characteristics measured with different frequencies are plotted in Supplementary Fig. 12c. The total gate capacitance (C_{gate}) consists of an electrostatic coupling capacitance $C_G=\varepsilon_0\varepsilon_r/t$ and quantum capacitance $C_q=\frac{2e^2\sqrt{n+n_0}}{\hbar v_F\sqrt{\pi}}$ as $\frac{1}{C_{gate}}=\frac{1}{C_G}+\frac{1}{C_q}$, where ε_0 is the vacuum permittivity, ε_r and t are the relative dielectric constant and the thickness of the quasi-2D amorphous carbon film sandwiched between the top and bottom electrodes, respectively, e is the elementary charge, n is the modulated carrier concentration, $\hbar$ is the reduced Planck constant, and $v_F \sim 10^8$ cm/s is the Fermi velocity of carriers in graphene. N is further correlated with the applied gate overdrive as $V_{GS}-V_{G,Dirac}=\frac{e}{C_G}n+\frac{\hbar v_F\sqrt{\pi n}}{e}$. Fitting to the measured capacitance-voltage curves (Supplementary Fig. 12d) enables us to extract ε_r, which agrees with what we obtained from metal/quasi-2D amorphous carbon film/silicon capacitors (Supplementary Fig. 12e), and n_0,

which again agrees quantitatively with the average of the number determined by fitting the transfer curves of the graphene transistors (Supplementary Fig. 12f).

Supplementary Note 7: Performance comparison of graphene transistors built with quasi-2D amorphous carbon films or h-BN as gate dielectrics.

The performance of the graphene transistors incorporating quasi-2D amorphous carbon films as the gate dielectrics is benchmarked against those employing h-BN, as summarized in Supplementary Table 2. Since the leakage current through nanometre-thick quasi-2D amorphous carbon films is much smaller (Fig. 3h), a much thinner dielectric thickness can be adopted to enable the operation of graphene transistors under a reduced gate-voltage range[17,25,26]. In fact, in most reports in literature, h-BN was only used as the interfacial layer to improve the interface with graphene, but a much thicker metal oxide was required beneath the h-BN to ensure the leakage current would not affect the graphene transistor operations[18,27-32]. The mobility of our graphene transistors employing quasi-2D amorphous carbon films as gate dielectrics is comparable to those built on CVD graphene and h-BN[17,25]. The mobility of devices built on exfoliated graphene is much higher with less defects in the graphene channel[26]. Both quasi-2D amorphous carbon films and h-BN can improve the effective mobility of graphene transistors by 2–3 times compared to bulk metal oxides[18,27-32], since they both form the better interface with the graphene with their dangling-bond-free surfaces. In addition to enabling more aggressively scaled dielectric thickness, compared to h-BN, the adoption of quasi-2D amorphous carbon films as gate dielectrics of graphene transistors also allows more precise thickness control due to its layer-by-layer deposition process (Fig. 1g and Supplementary Fig. 5), with better scalability into wafer scale (Fig. 1a and Supplementary Fig. 1).

Supplementary Note 8: Properties of 2D MoS_2 channel.

Large-area, single-layer MoS_2 with thickness around 7 Å (Supplementary Fig. 13a and 13b) grown by CVD was utilized as the 2D semiconductor channel for integration with quasi-2D amorphous carbon films and silicon nitride as gate dielectrics to realize field-effect transistors. The Raman spectrum shows two characteristic modes corresponding to the in-plane (E^1_{2g}) and

out-of-plane (A_{1g}) vibrations. The difference in their frequency is about 19 cm^{-1}, suggesting a monolayer of MoS_2 (Supplementary Fig. 13c)[33]. The photoluminescence spectrum shows a strong peak at 1.85 eV, which is associated with the direct-gap transition at K point for monolayer MoS_2 (Supplementary Fig. 13d)[34]. The XPS spectra (Supplementary Fig. 13e and 13f) show the Mo $3d_{3/2}$, Mo $3d_{5/2}$, S $2s$, S $2p_{1/2}$, and S $2p_{3/2}$ peaks, giving a Mo to S ratio of 1:1.9[35].

Supplementary Note 9: Performance comparison of 2D MoS_2 transistors built with quasi-2D amorphous carbon films, h-BN, or 3D bulk oxides as gate dielectrics.

The performance of the 2D MoS_2 transistors incorporating quasi-2D amorphous carbon films as the gate dielectric is benchmarked against those employing h-BN and various bulk metal oxides, as summarized in Supplementary Table 3. Quasi-2D amorphous-carbon-film gate dielectric allows the adoption of an ultrathin film thickness to ensure low device operating voltage range[36-45], and potentially lower gate-leakage current density (Fig. 3h), with precisely controlled film thickness (Fig. 1g and Supplementary Fig. 5) and excellent uniformity on wafer scale (Fig. 1a and Supplementary Fig. 1), compared to h-BN[36-39]. It also helps the 2D MoS_2 transistors to achieve a relatively high effective mobility (>15 $cm^2 \cdot V^{-1} s^{-1}$) among those built on monolayer MoS_2 grown by CVD[39-44]. The subthreshold swing is comparable to that of devices using h-BN gate dielectric with their similar capability to form dangling-bond-free interface with the 2D semiconductor channel[36-39], but superior to those built on most bulk metal oxides[40-45]. Those devices adopting $SrTiO_3$ and CaF_2 exhibited better performance, but the strong frequency and temperature dependence of permittivity of perovskite ceramics and ionic crystals could create reliability potential problems for electronic applications[46,47].

Supplementary Note 10: Performance benchmark of memristors employing quasi-2D amorphous carbon bilayer with those built with 3D oxides or 2D dielectrics/semiconductors.

The performance of Pt/quasi-2D amorphous carbon bilayer/Ag memristors is compared with those built with other 2D materials (h-BN, MoS_2, graphene oxide)[48-51] or 3D bulk oxides (oxygenated amorphous carbon, HfO_2, Al_2O_3, TaO_2, TiO_2)[52-56]. The device key performance metrics and characteristics, including the switching voltages (Set and Reset) and their variability,

device size and film thickness, device endurance and data retention, the contrast between the on- and off-states, and the switching speed and energy consumption per transaction, are summarized in Supplementary Table 4. Memristors employing quasi-2D amorphous carbon films as the ion-transport media demonstrate very low operating voltages together with low variability with their intrinsic ultrathinness and confined filament-formation pathways (Fig. 8h), without sacrificing the data retention or the device endurance. In pulse measurement, since the formation of a conductive pathway across the atomically thin quasi-2D amorphous carbon bilayer requires the movement of a very small number of Ag atoms across a short distance, the device can be operated at a fast-switching speed < 20 ns with low energy consumption down to 20 fJ per transaction (Fig. 8i). Their energy-delay product is therefore among the best values ever reported for resistive memory devices.

Supplementary Note 11: Finding the proper concentration to achieve film thickness down to 1–2 atomic layers.

To achieve film thickness down to 1–2 atomic layers, it is critical to control the concentration of the carbon-dot precursors. If the concentration is too low, e.g., 0.01 $mg \cdot mL^{-1}$, the spin-casting process produced a sub-monolayer of carbon-dot precursors with microscopic holes visible in the AFM image (Supplementary Fig. 17a). At the optimized concentration of 0.05 $mg \cdot mL^{-1}$, the film is continuous with a thickness of ~0.8 nm and surface roughness comparable to that of the thermal SiO_2/Si substrate (Supplementary Fig. 17b and Fig. 1f) as measured by AFM, and features low density of pin-hole type defects (Supplementary Fig. 6). However, if the concentration is too high, e.g., 0.1 $mg \cdot mL^{-1}$, the thickness of the deposited films will be increased to above 1–2 atomic layers (Supplementary Fig. 19c). Moreover, the arrangement of the carbon dots was disturbed with the non-planar stacking or tilting of some deposited dots, which not only increases the film surface roughness but also reduces the packing density of carbon atoms, as evident from its much lower Raman intensity than that of a film prepared by the layer-by-layer deposition process, despite their similar thickness of 1.3–1.4 nm as measured by AFM (Supplementary Fig. 19d and 19e).

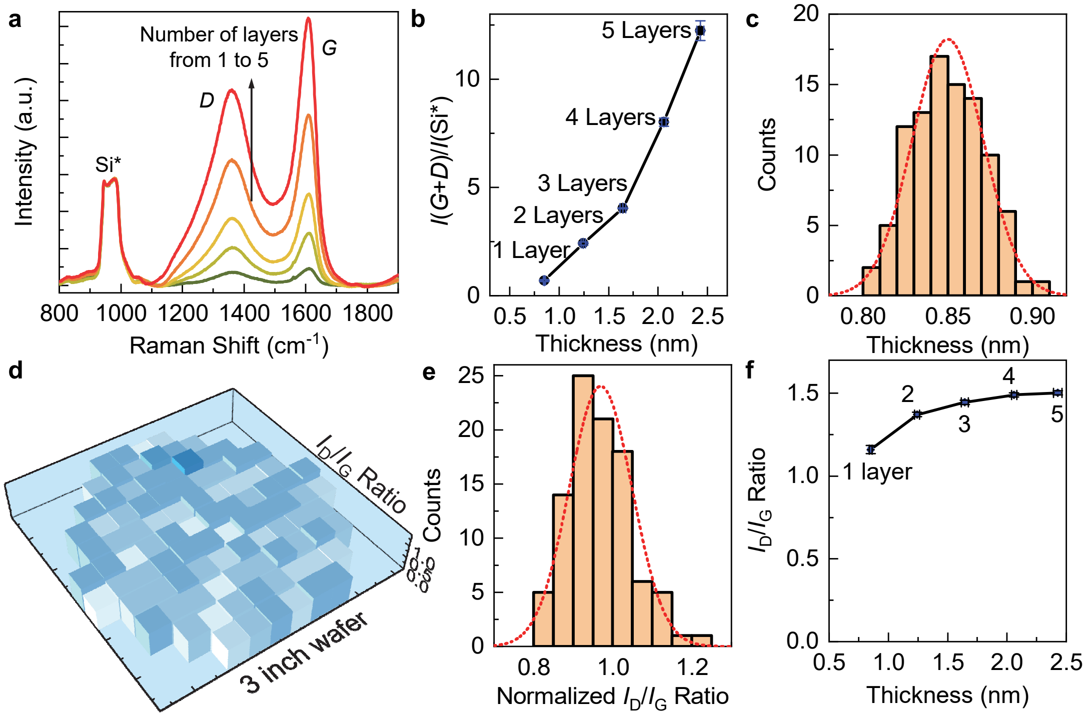

Supplementary Figure 1| Raman characterizations of quasi-2D amorphous carbon. **a**, Raman spectra taken for films with different numbers of layers. *D*: disorder band of carbon-based materials; *G*: in-plane vibrations of sp^2 bonded carbon; Si*: second-order optical phonon peak of silicon. **b**, The integral intensity of the (*D*+*G*) bands of the quasi-2D amorphous carbon normalized to the intensity of the Si* peak as a function of the film thickness measured by AFM. **c**, Histogram showing the distribution of film thickness mapped across the 3-inch wafer covered with an ultrathin quasi-2D amorphous carbon, with the film thickness determined from the integral Raman intensity based on its correlation with the film thickness plotted in part **b**. Red dashed line represents a Gaussian fit to the data. **d**, Spatial mapping of the measured I_D/I_G ratio for ultrathin quasi-2D amorphous carbon across the 3-inch wafer. **e**, Histogram of the spatial mapping data in part **d**, showing a normalized standard deviation down to 8%. The red dashed line represents a Gaussian fit to the data. **f**, The *D* band to *G* band intensity ratio for quasi-2D amorphous carbon films with different numbers of layers as a function of the film thickness measured by AFM. Error bars represent the standard deviation.

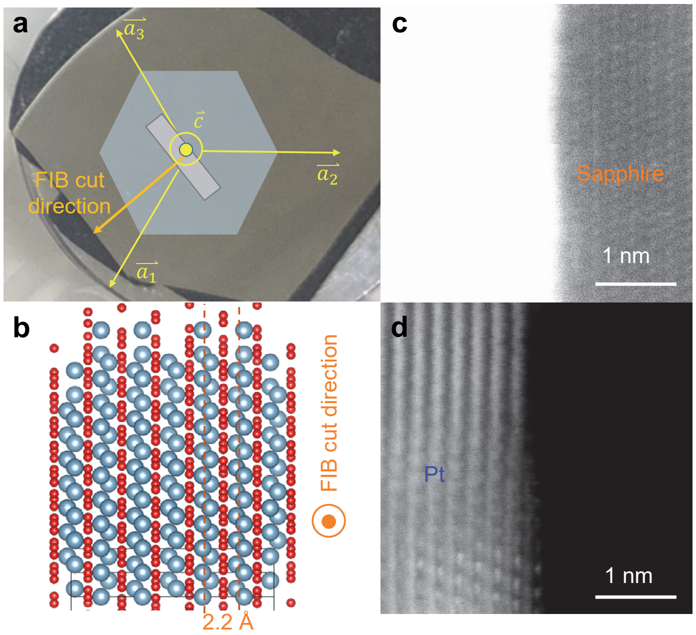

Supplementary Figure 2| Sample preparation and the processing of high-resolution STEM micrographs to determine the thickness of ultrathin quasi-2D amorphous carbon. **a**, Schematic illustrating the FIB-slicing direction $\left[5\,\bar{4}\,\bar{1}\,0\right]$ relative to the *a* and *c* axes of sapphire. **b**, Schematic showing the 2.2 Å distance between two neighbouring aluminium planes with an oxygen plane sandwiched in between. **c-d**, Brightness-adjusted images showing the sapphire lattice (part **c**) and Pt lattice (part **d**) before overlay to produce Fig. 1d.

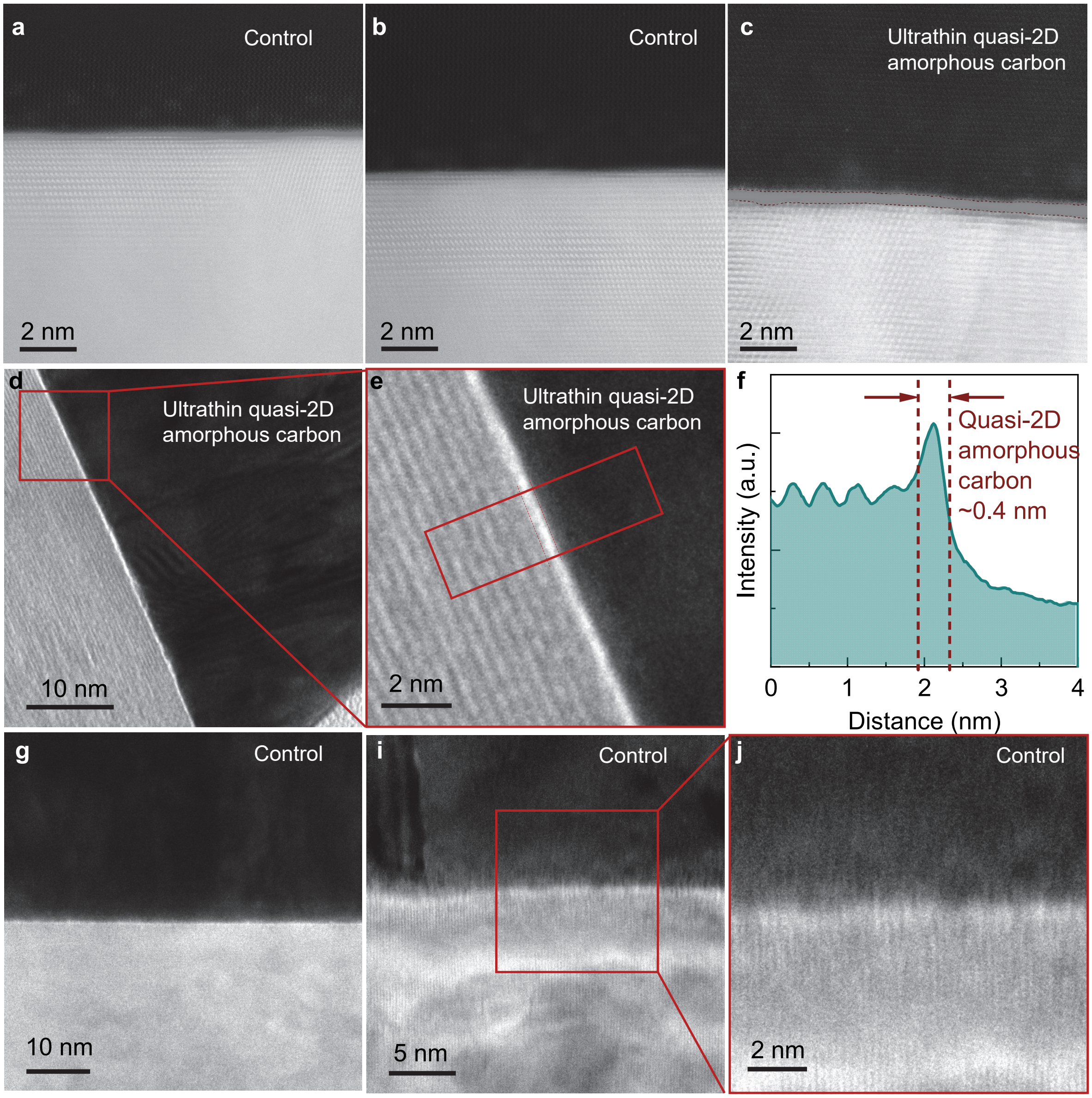

Supplementary Figure 3| Comparison with the cross-sectional STEM/TEM micrographs of Pt/sapphire control samples. a-b, Cross-sectional STEM images of Pt deposited directly on a *c*-cut sapphire wafer, taken at two different locations. **c**, Cross-sectional STEM image of sapphire/quasi-2D amorphous carbon/Pt, showing the presence of the ultrathin carbon film sandwiched in between, in clear comparison with the control samples shown in frame a-b. **d-f**, Cross-sectional phase-contrast TEM images of sapphire/quasi-2D amorphous carbon/Pt under low (part **d**) and high (part **e**) magnifications, with the associated contrast intensity profile (part **f**) clearly showing the presence of the ultrathin carbon film sandwiched in between. **g-i**, Cross-sectional phase-contrast TEM images of the sapphire/Pt control samples under low (part **g**), intermediate (**h**), and high (part **i**) magnifications, showing that the direct contact between the sapphire and the Pt lattice. The slightly blurring at the interface is likely caused by the surface roughness in the projection depth.

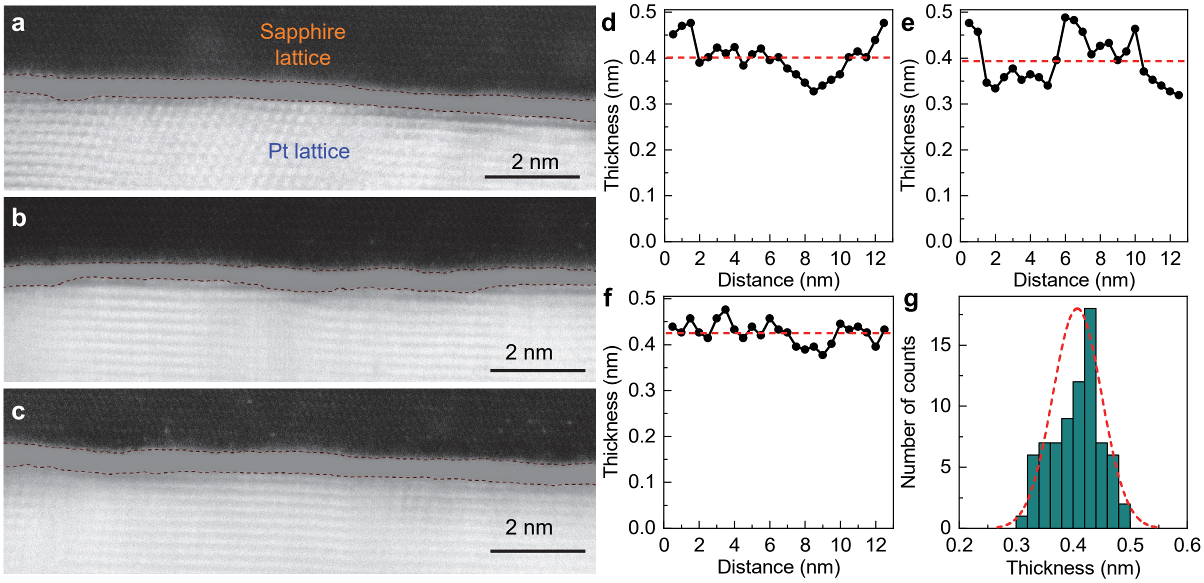

Supplementary Figure 4| Thickness uniformity of ultrathin quasi-2D amorphous carbon. **a-f**, Low-magnification cross-sectional STEM images of ultrathin quasi-2D amorphous carbon formed on a sapphire substrate (**part a-c**), taken at different locations across the wafer, and their correesponding thickness profiles (**part d-f**). The red dashed lines mark the average. **g**, Histogram showing the film thickness distribution. The red dashed line represents a Gaussian fitting to the data.

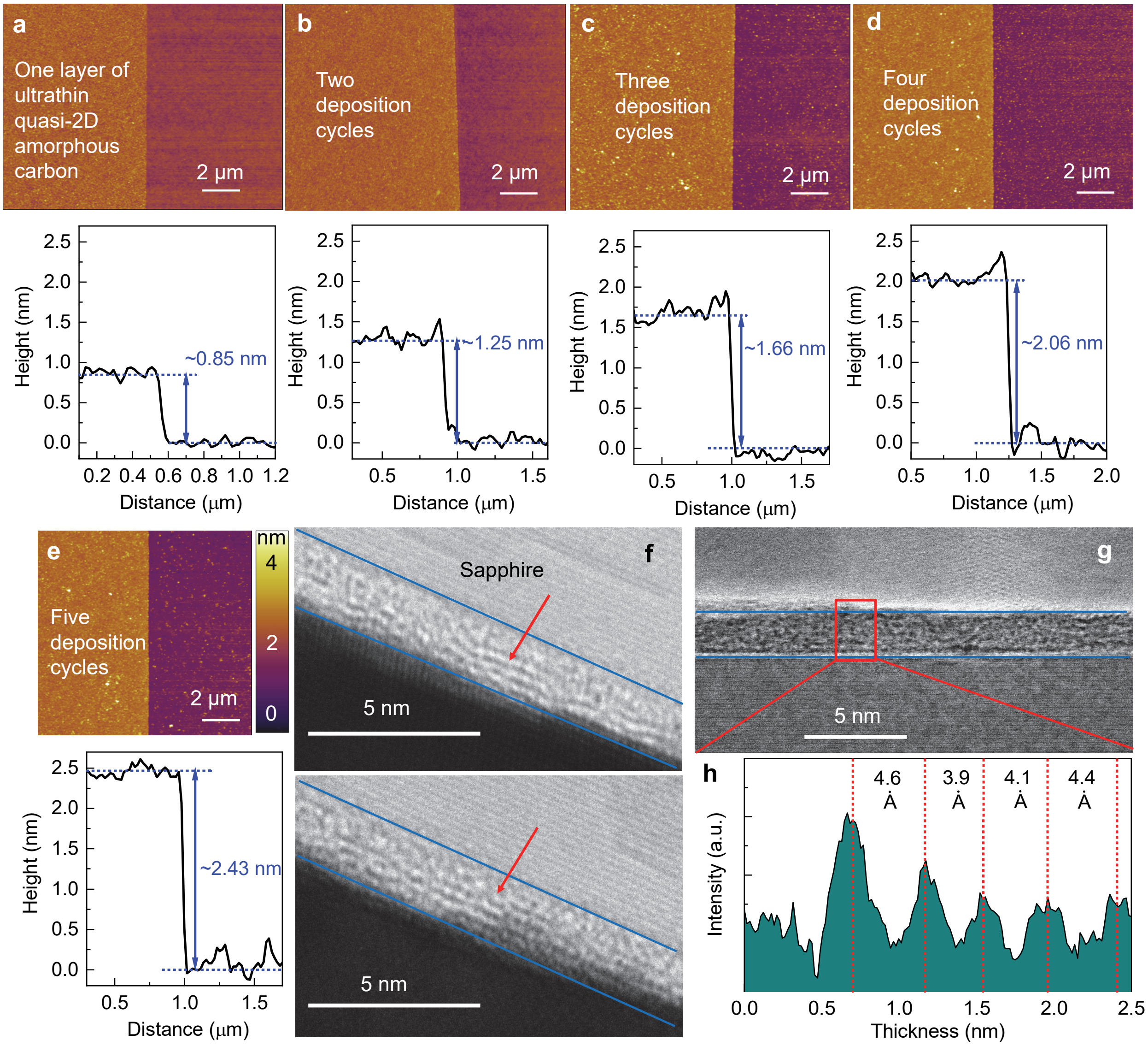

Supplementary Figure 5| Multilayered quasi-2D amorphous carbon films deposited in a layer-by-layer fashion. **a-e,** AFM images (top frames) and line-cut profiles (bottom frames) of quasi-2D amorphous carbon patterned to generate a step height. Part **a** corresponds to a single deposition corresponding to an ultrathin film with thickness down to 1–2 atomic layers. Part **b-e** represent films formed by repeating the spin-cast deposition and annealing cycles for one (part **b**), two (part **c**), three (part **d**), up to four (part **e**) additional times. **f-g**, Cross-sectional TEM (part **f**) and STEM (part **g**) images of the five-layered film shown in part **e**. As visual guide, blue solid lines mark the positions of interfaces, and red arrows point to the atomic layers within the deposited films. **h**, Contrast intensity profile. Red dotted lines mark peak positions corresponding with atomic layers, indicating interlayer spacings ~0.4 nm.

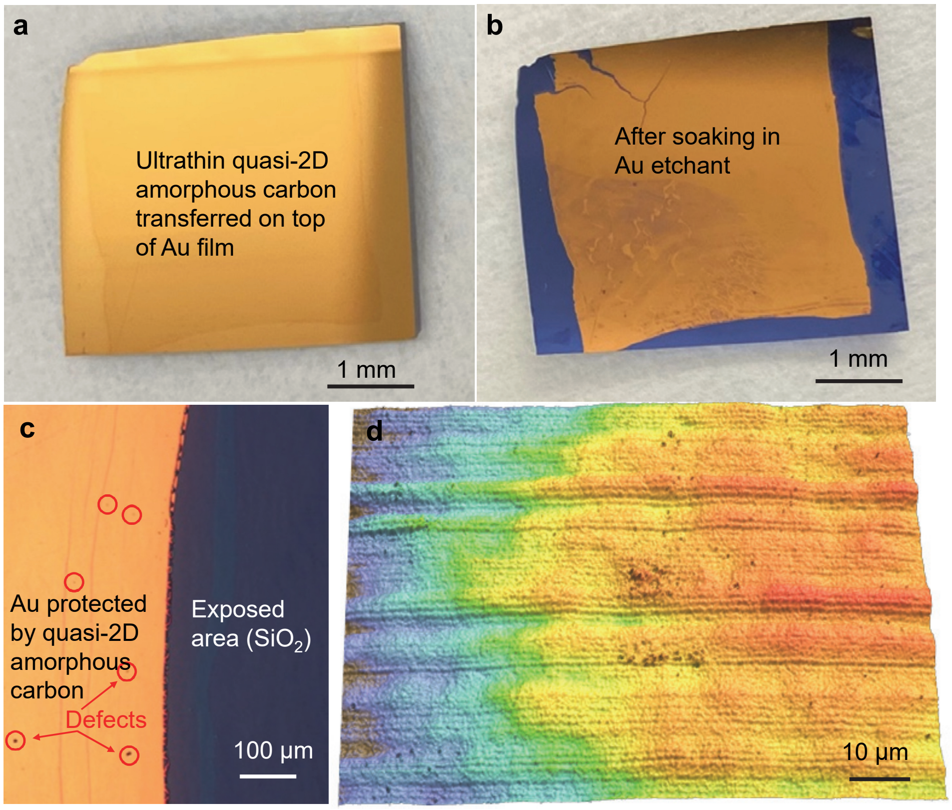

e Sample # Area=7500 µm^2	1	2	3	4	5	6
Number of defects	158	173	71	164	112	84
Defect density	(17±5)x10^3 per mm^2					

Supplementary Figure 6| Pin-hole defect density of transferred ultrathin quasi-2D amorphous carbon nanomembrane with thickness down to 1–2 atomic layers. a-b, Optical images of a sample with a transferred ultrathin quasi-2D amorphous carbon covering a substrate of Au (200 nm)/SiO_2 (90 nm)/Si before (part **a**) and after (part **b**) immersing in Au etchant. **c**, Magnified view showing the dark spots corresponding to pin-hole defects in the transferred film. **d**, 3D laser scanning microscopic image to show etched pits. **e**, Table summarizing defect densities measured at 5 locations across the wafer.

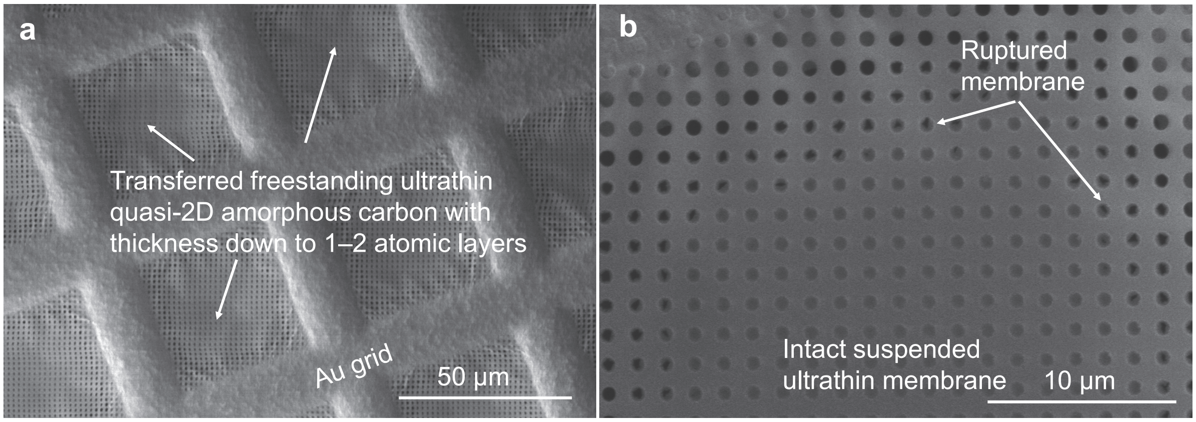

Supplementary Figure 7| SEM images of the freestanding ultrathin quasi-2D amorphous carbon with thickness down to 1–2 atomic layers suspended on a holey-carbon TEM grid. **a,** Low-magnification SEM micrograph showing the uniform coverage of a freestanding ultrathin quasi-2D amorphous carbon with thickness down to 1–2 atomic layers over large area. **c,** Zoom-in image showing the suspended membrane is intact in the centre but has high probability to rupture near the edge.

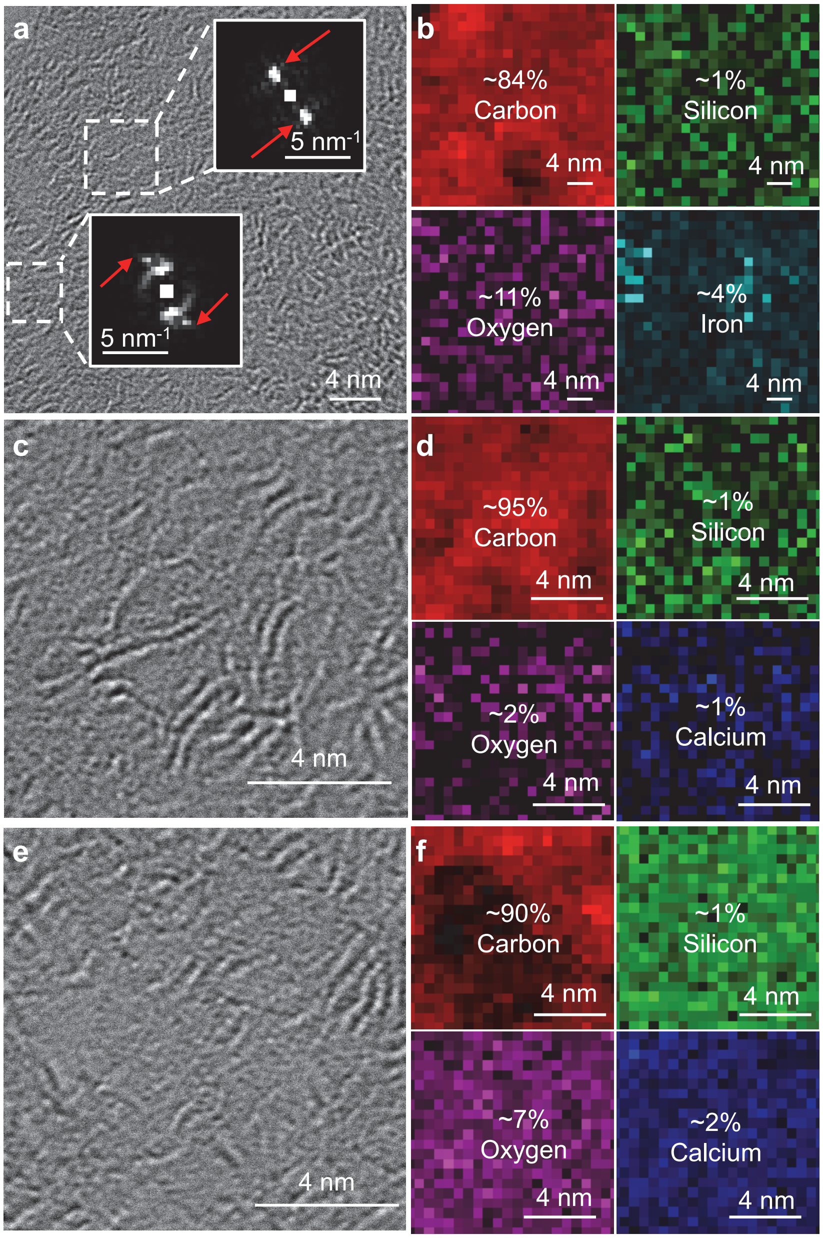

Supplementary Figure 8| STEM images (part a, c, and e) and associated EELS mappings (part b, d, and e) of a freestanding, ultrathin quasi-2D amorphous carbon nanomembrane with thickness down to 1–2 atomic layers, taken at separate locations. In part **a**, white dashed lines serve as visual guide to mark the boundaries of some selected regions with regular atomic patterns and the insets show their corresponding fast Fourier transforms. Red arrows serve as visual guide pointing to the Bragg spots.

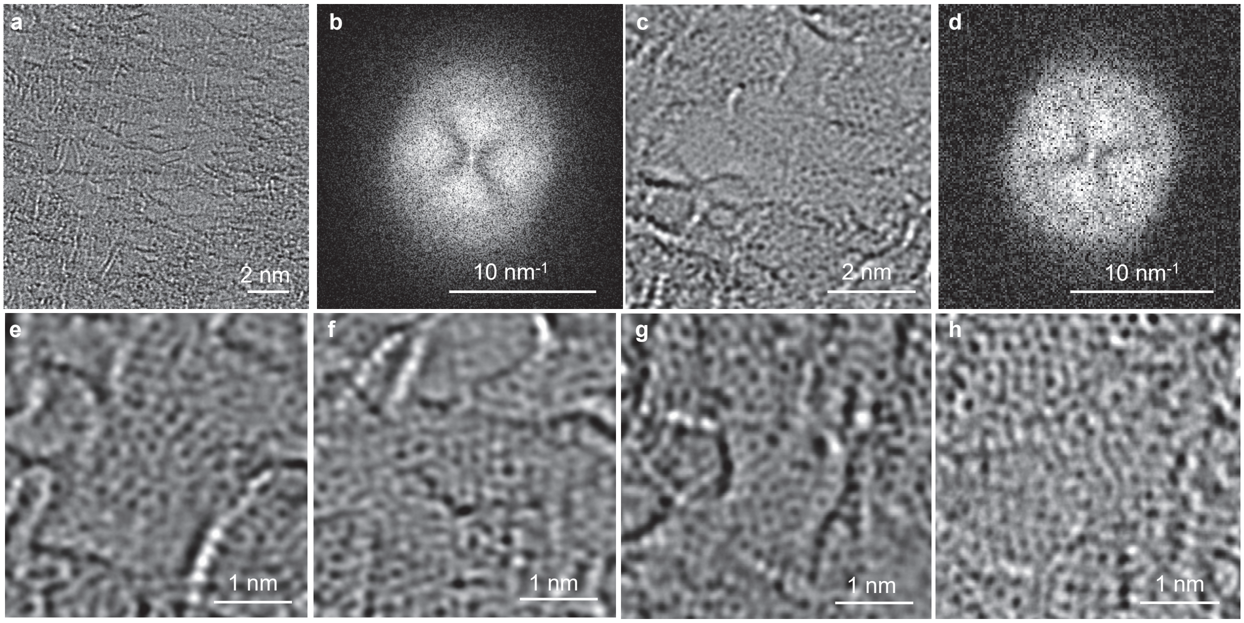

Supplementary Figure 9| STEM images of ultrathin quasi-2D amorphous carbon freestanding nanomembrane with thickness down to 1–2 atomic layers at randomly selected sites across the TEM grid. a-d, 15×15 nm^2 (part **a**) and 7×7 nm^2 (part **c**) STEM images of ultrathin quasi-2D amorphous carbon and their associated fast Fourier transform patterns (part **b** and part **d**) exhibiting the characteristic diffuse halo for amorphous materials, consistent with the SAED pattern shown in Fig. 2a. **e-h**, Aberration-corrected high-resolution STEM images at random sites.

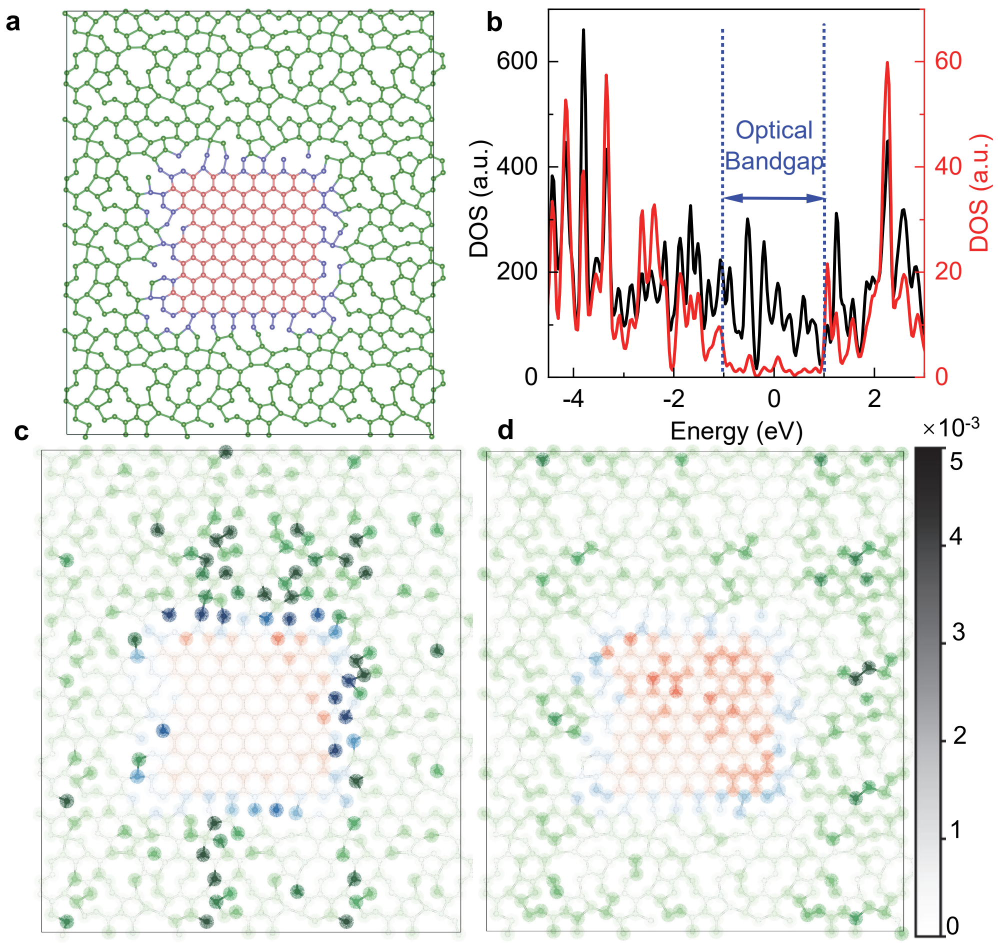

Supplementary Figure 10| Electronic structure of idealized 2D amorphous carbon simulated by DFT. a, Top view of the unit cell used in the simulation. Carbon atoms in the nanocrystallite, the fully amorphous Zachariasen regions, and the interface in between are coloured in red, green, and blue, respectively. **b,** Density of states (DOS) projected on 2D amorphous carbon (black solid line, left axis) and embedded nanocrystallite (red dashed line, right axis), respectively. The blue dashed lines serve as visual guide to mark the simulated effective optical bandgap. **c-d**, Mapping of the atom-projected DOS at the Fermi energy (part **c**) and 4 eV below the Fermi energy (part **d**), for each atom, overlaid with the unit-cell atomic structure. The colour represents the different types of carbon atoms as in part **a**, and each atom's normalized contribution to the total DOS is quantitatively correlated with the colour darkness as illustrated in the grayscale colour bar whose upper end corresponds to the 98th percentile of DOS contribution. This confirms the localization of states in the "optical bandgap" range (see part **b**), which affects the optical absorption strength.

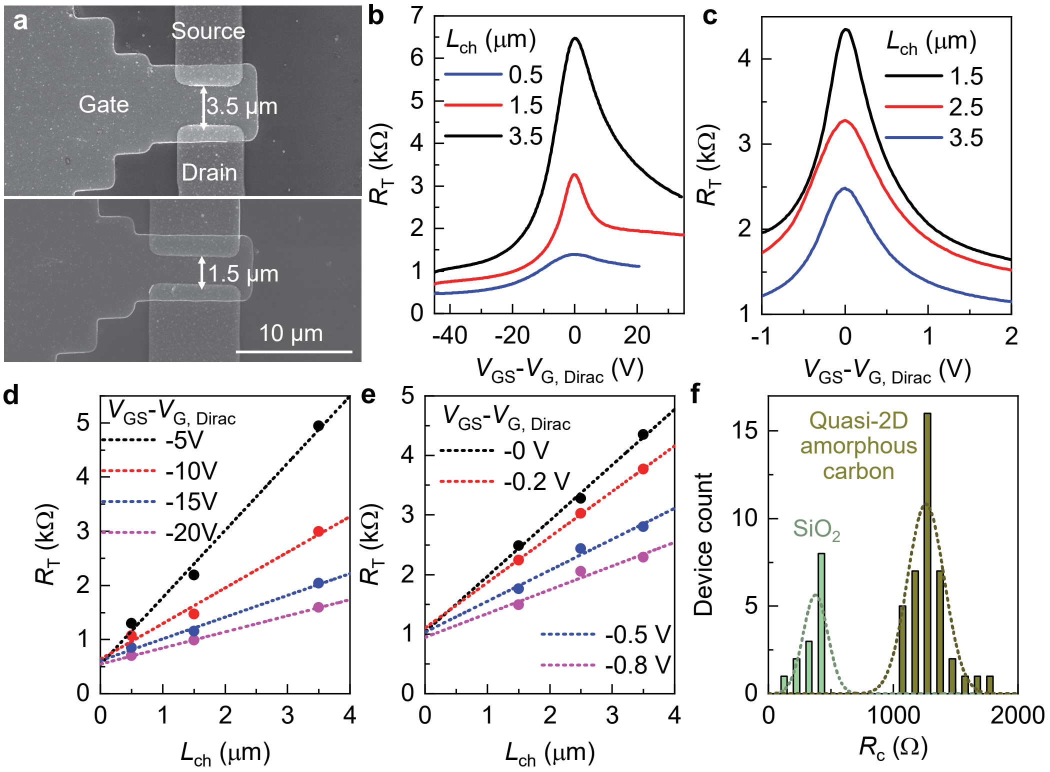

Supplementary Figure 11| Contact resistance of graphene transistors extracted using the transmission-line method. **a,** SEM micrographs of top-gated graphene transistors with long (3.5 μm, top frame) and short (1.5 μm, bottom frame) L_{ch}. **b**, R_T modulated by the gate overdrive voltage for graphene bottom-gated transistors with identical W of 3.5 μm and L_{ch} varied from 3.5 μm (black), 1.5 μm (red), to 500 nm (blue) from top to bottom. **c**, R_T modulated by the gate overdrive voltage for graphene top-gated transistors with identical W of 3.5 μm and L_{ch} varied from 3.5 μm (black), 2.5 μm (red), to 1.5 μm (blue) from top to bottom. Applied V_{DS} is 0.2 V. **d-e**, R_T as a function of L_{ch} under different gate overdrives to extract R_c for bottom-gated (part **d**) and top-gated (part **e**) graphene transistors. Dashed lines are linear fittings to the data. **f**, Comparison of the R_c extracted by the fitting method from bottom-gated graphene transistors using 90 nm SiO_2 as gate oxide (green) with those from top-gated devices employing quasi-2D amorphous carbon films as gate dielectric (dark yellow). Dotted lines represent Gaussian fittings to the data, where the averages agree well with the values extracted by the transmission-line method shown in part **d** and **e**.

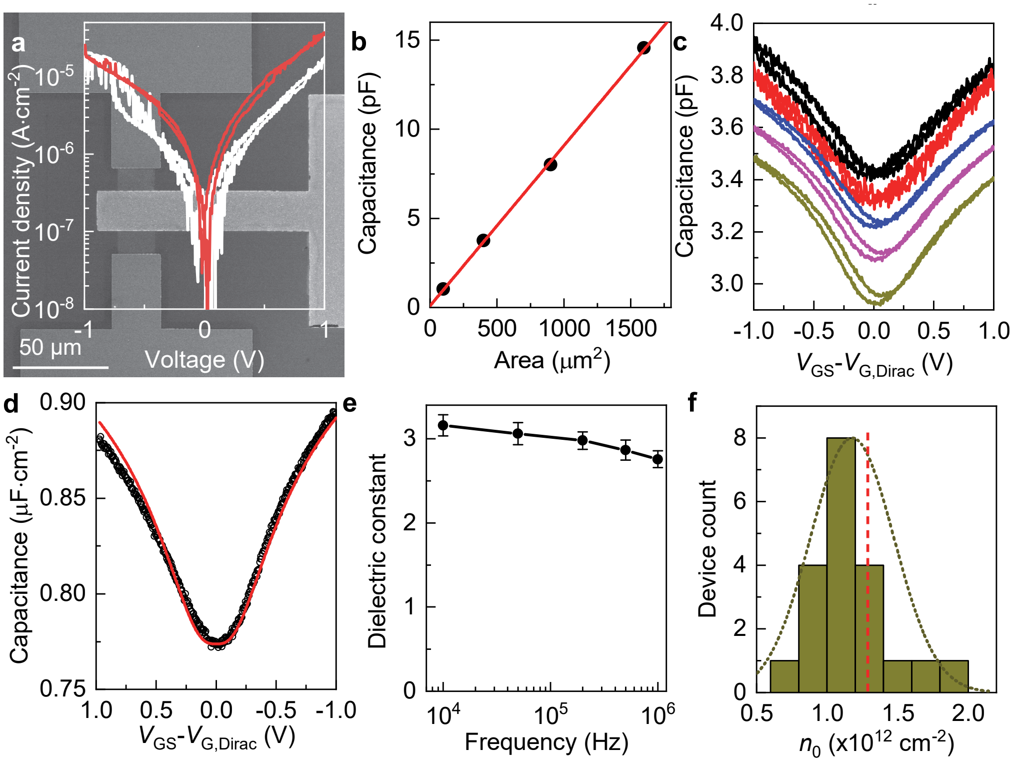

Supplementary Figure 12| Graphene/quasi-2D amorphous carbon/metal capacitors. **a,** SEM micrograph showing the fabricated graphene/five-layers of ultrathin quasi-2D amorphous carbon/metal capacitors. The graphene is contacted with Cr/Au electrodes. Inset: Leakage-current density as a function of bias applied between the top and bottom electrodes for capacitors with the dimension of 20 μm by 20 μm (white) and 40 μm by 40 μm (red), respectively. **b**, Capacitance measured under 1 V bias as a function of the capacitor area. The red solid line is the linear fitting to the data. **c**, Capacitance-voltage curves measured with the frequency varied from 10 kHz (black), 50 kHz (red), 200 kHz (blue), 500 kHz (magenta), to 1 MHz (dark yellow) from top to bottom, showing small hysteresis. **d**, Experimental plot (500 kHz, black circles) and model fit (red solid line) of the capacitance as a function of the gate overdrive. The extracted induced residual charged impurity concentration n_0 is $1.3\text{x}10^{12}$ cm^{-2} and ε_r is 2.9. **e**, Frequency dependence of the extracted ε_r of quasi-2D amorphous carbon films (five layers, thickness ~2.4 nm) evaluated between 10^4 and 10^6 Hz. Error bars represent s.d.. **f**, Histogram showing n_0 extracted from the transfer curves of top-gated graphene transistors by fitting. The dotted line represents a Gaussian fit to the data. The red dashed line serves as a visual guide to mark the position of n_0 corresponding to value determined independently from the capacitance-voltage curve shown in part **d**.

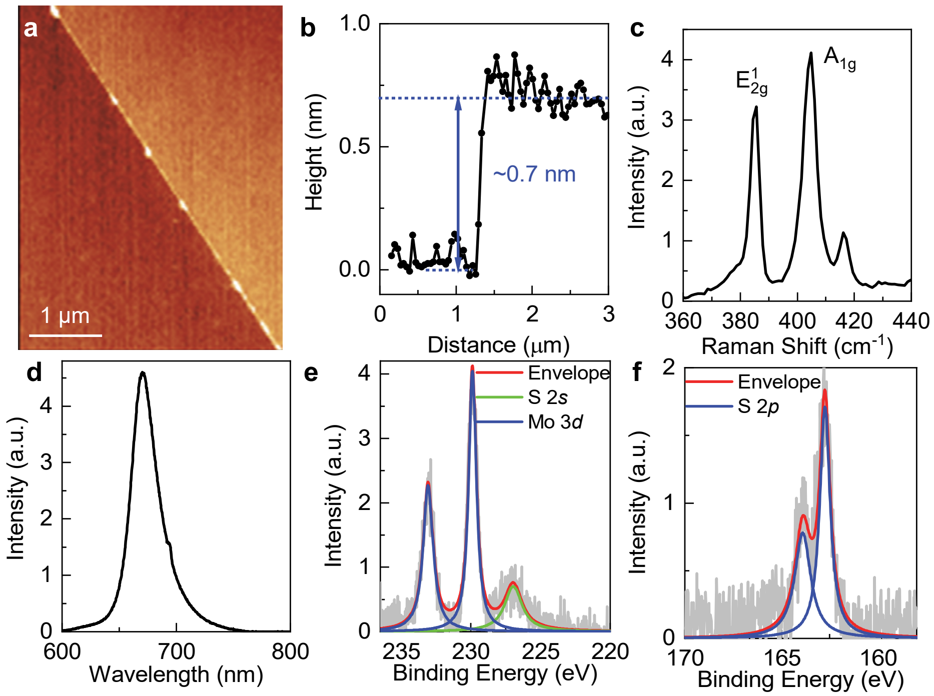

Supplementary Figure 13| Material characteristics of the MoS_2 monolayer channel. **a,** AFM image of 2D MoS_2 patterned to generate an edge by photolithography and etching. **b**, The linecut profile showing a step height ~0.7 nm corresponding with the MoS_2 film thickness. **c**, Raman spectrum showing the characteristic in-plane (E^1_{2g}) and out-of-plane (A_{1g}) vibrational modes. **d**, Photoluminescence spectrum showing a strong peak at 1.85 eV (670 nm). **e-f**, High-resolution XPS spectra of MoS_2, with the fitted curves showing the Mo 3*d* (blue in part **e**), S 2*s* (green in part **e**), and S 2*p* (blue in part **f**) peaks and the overall envelope (red).

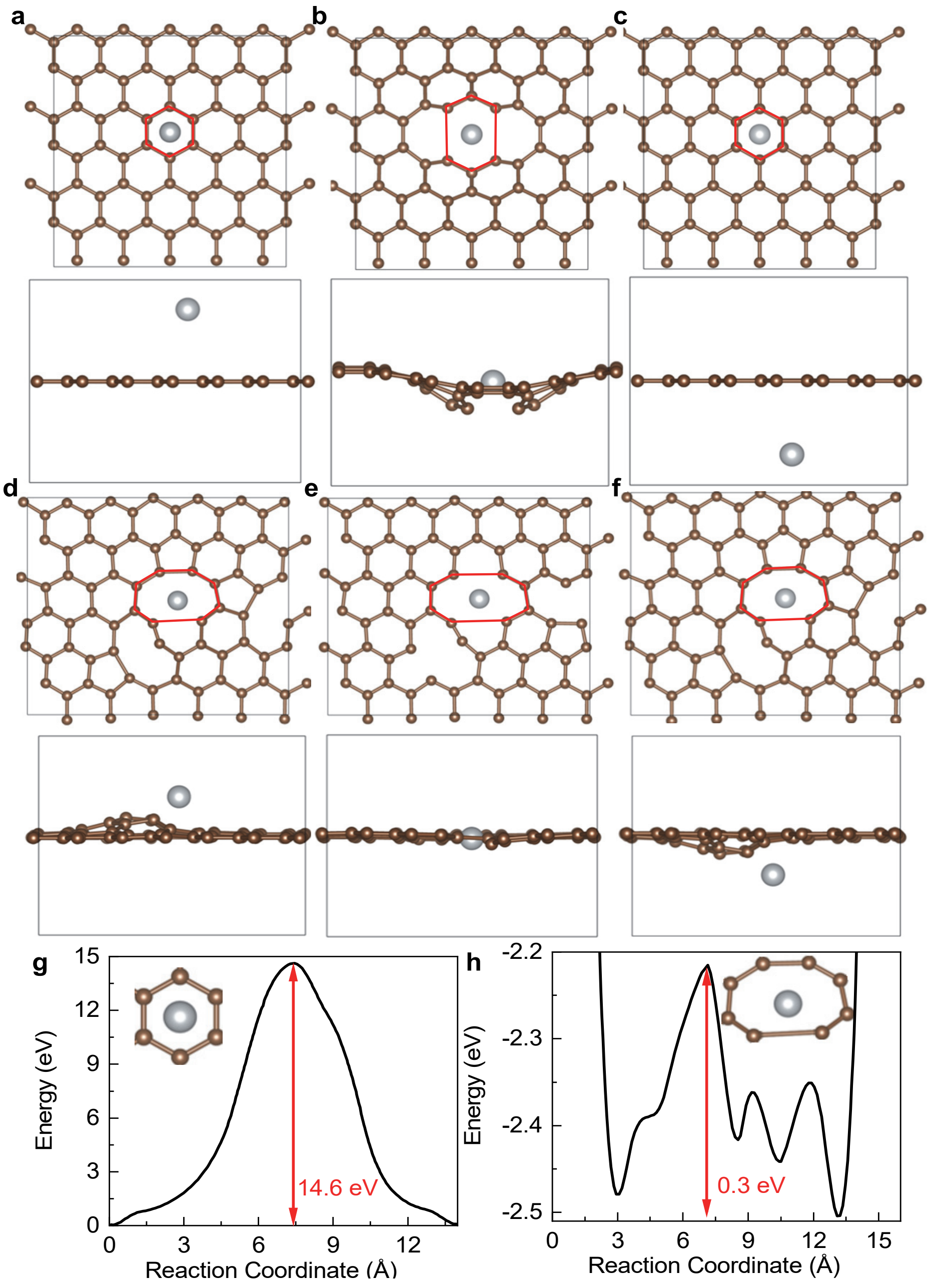

Supplementary Figure 14| DFT simulation of the diffusion of a Ag ion through carbon rings. a-c, Top views (top frames) and side views (bottom frames) of the initial (part **a**), intermediate (part **b**), and final (part **c**) states of the pathway for a Ag ion to diffuse through a benzene ring highlighted in red. **d-f,** Top views (top frames) and side views (bottom frames) of the initial (part **d**), intermediate (part **e**), and final (part **f**) states of the pathway for a Ag ion to diffuse through an octagonal carbon ring highlighted in red. **g-h,** Simulated reaction coordinate for a Ag ion through six-membered (part **g**) and eight-membered (part **h**) carbon rings, respectively. Red arrowed lines mark the height of the energy barrier.

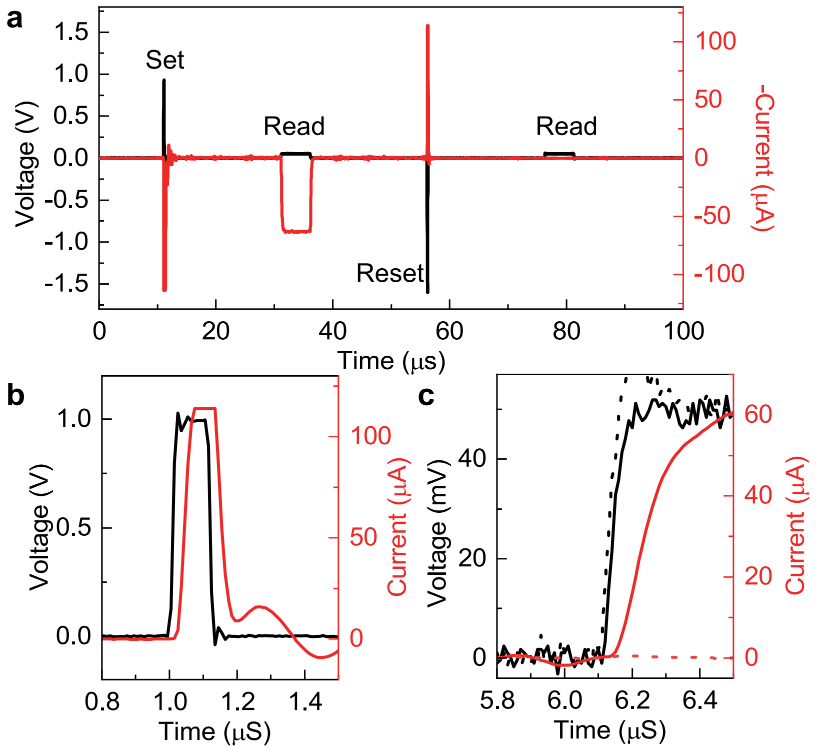

Supplementary Figure 15| Switching of memristors employing quasi-2D amorphous carbon bilayers as the ion-transport media with voltage pulses. **a**, Resistive switching of a Pt/quasi-2D amorphous carbon/Ag memristor going through the programming sequence of set (1V, 100 ns), read (0.05 V, 5 µs), reset (-1.5 V, 100 ns), and read (0.05 V, 5 µs). **b**, Applied voltage (left axis, black) and the response current (rights axis, red) *versus* time plots showing that switching of the device with 100 ns/1 V set-voltage pulse under a 100 times higher compliance current of 100 µA still gives a similar t_{set} of 20 ns. **c**, The leading edge of the applied voltage (left axis, black) and the response current (right axis, red) *versus* time plots of the device under 0.05 V read voltage pulse after the set (solid lines) and reset (dashed lines) operations, again exhibiting the similar ~20 ns delay as in the set operation shown in part **b**. Therefore, the observed ~20 ns delay is likely dominated by the parasitic capacitance rather than the device intrinsic switching speed.

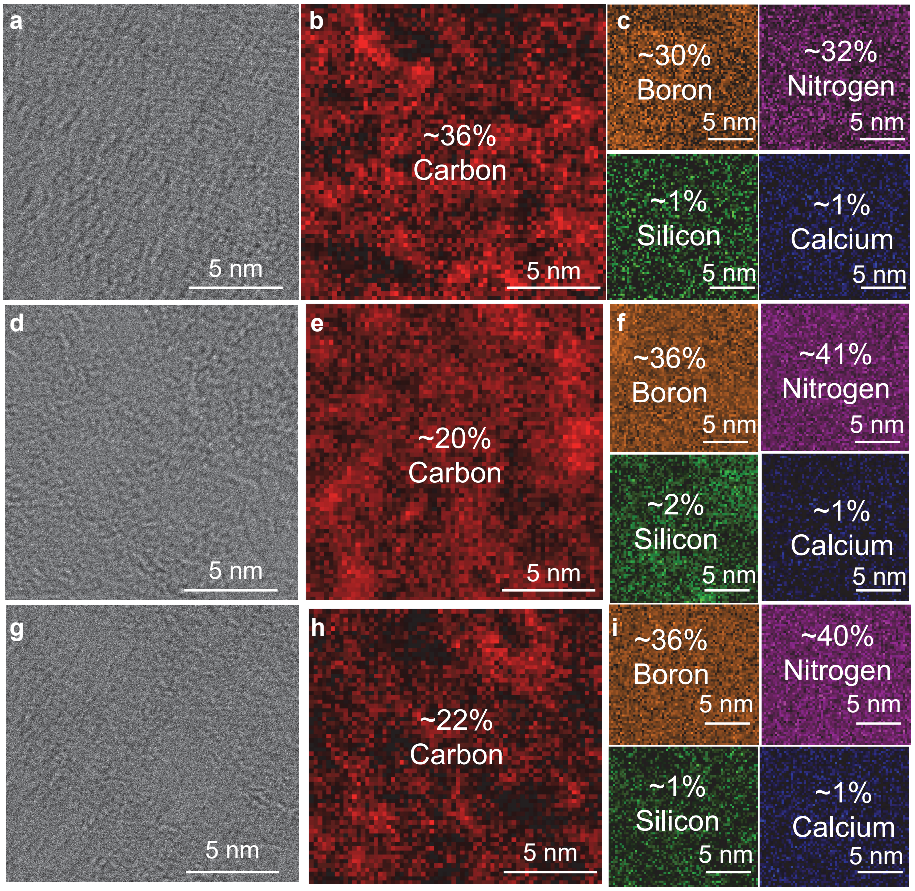

Supplementary Figure 16| STEM images (part a, d, and g) and the associated EELS mappings (part b, c, e, f, h, and i) of sub-monolayer carbon-dot precursors deposited on a h-BN freestanding membrane support at three different locations.

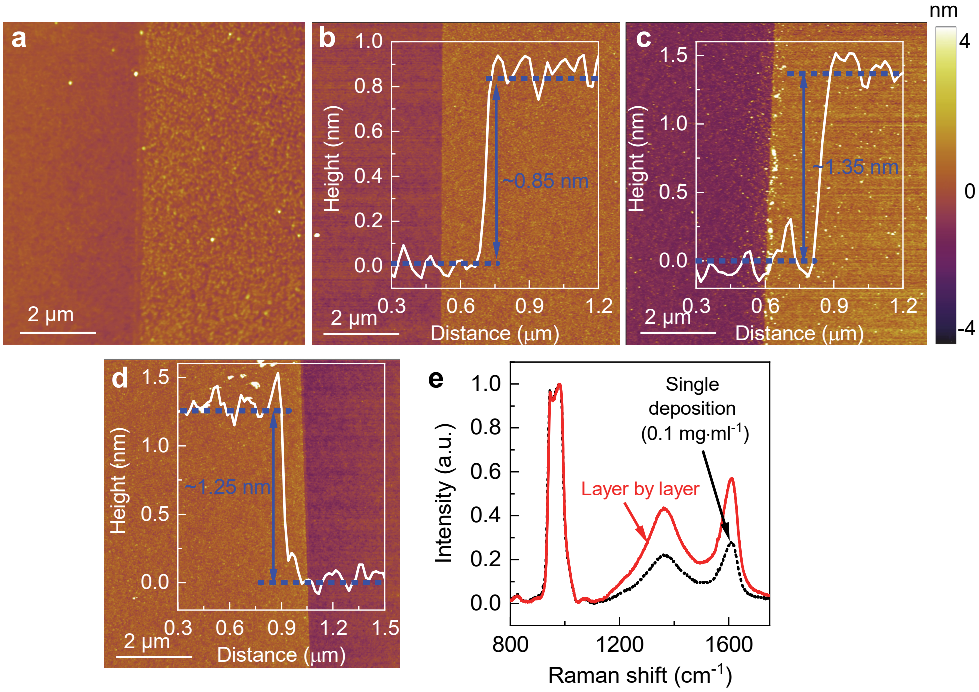

Supplementary Figure 17| Spin-coating deposition of carbon-dot precursors with different concentrations. a-d, AFM images and line-cut profiles (inset) of spin-casted carbon dots on SiO_2/Si wafers. Part **a-c** correspond to films generated by a single coating from the carbon-dot dispersions with concentrations varied from 0.01 (part **a**) to 0.05 (part **b**) and 0.1 (part **c**) mg·mL^{-1}. Part **d** corresponds to a film formed by performing the deposition and coalescence cycles twice from a carbon-dot dispersion with a concentration of 0.05 mg·mL^{-1}. **e**, Raman spectra of the amorphous carbon films with almost identical thickness but formed by either a layer-by-layer deposition (solid red line) or a single deposition of a more concentrated precursor solution (black dotted line).

Materials	Interlayer Spacing (nm)	Modulus (GPa)	Bandgap (eV)	Dielectric Constant	Dielectric Strength ($MV \cdot cm^{-1}$)	Resistivity ($\Omega \cdot cm$)
Quasi-2D amorphous Carbon	~0.4	400±100	3.5	~3	22±2	$>10^9$
h-BN	0.33–0.34	200–810	~6	2.9–3.8	4–21	$\sim 5\times 10^{10}$
References for h-BN	[6,7]	[8,9]	[10,11]	[12,13]	[14-19]	[20,21]

Supplementary Table 1| Comparison of the physical properties of quasi-2D amorphous carbon and h-BN.

Materials	Role	Gate dielectric thickness (nm)	Channel materials	Mobility ($cm^2 \cdot V^{-1} s^{-1}$)	μ improvement (times, w/wo)	Operation voltage (V)	Ref
Quasi-2D amorphous Carbon	Gate Dielectric	1.6	CVD Graphene	~2500	>2	-1.5 to 1.5	This Work
CVD h-BN	Gate Dielectric	15	CVD Graphene	2141	-	0 to 8	[17]
Exfoliated h-BN	Gate Dielectric	19	CVD Graphene	2300	-	-3 to 3	[25]
Exfoliated h-BN	Gate Dielectric	8.5	Exfoliated Graphene	10713	-	-2 to 2	[26]
CVD h-BN	Interfacial Dielectric	0.5 nm h-BN +90 nm SiO_2	CVD Graphene	6213	~1.71	-30 to 60	[27]
CVD h-BN	Interfacial Dielectric	5 nm h-BN +thick SiO_2	CVD Graphene	3400	>3.5	0 to 60	[28]
CVD h-BN	Interfacial Dielectric	2 nm h-BN +90 nm SiO_2	CVD Graphene	~3000	~2	-15 to 30	[29]
CVD h-BN	Interfacial Dielectric	2 nm h-BN +300 nm SiO_2	CVD Graphene	6850	~2.5	-40 to 40	[30]
CVD h-BN	Interfacial Dielectric	4 nm h-BN +300nm SiO_2	CVD Graphene	~6500	>2	-60 to 60	[31]
CVD h-BN	Interfacial Dielectric	12 nm h-BN +300nm SiO_2	Exfoliated Graphene	~7500	~1.23	-25 to 25	[18]
Exfoliated h-BN	Interfacial Dielectric	60 nm h-BN +300 nm SiO_2	CVD Graphene	~7000	~2	-50 to 50	[32]

Supplementary Table 2| Comparison of the performances of graphene transistors built with quasi-2D amorphous carbon films or h-BN as the gate dielectrics.

Gate Dielectric Materials	Device Structure	Dielectric Thickness (nm)	Channel Materials	MoS_2 Thickness (nm)	Mobility ($cm^2 \cdot V^{-1} s^{-1}$)	Subthreshold Swing ($mV \cdot Dec^{-1}$)	Operating Voltage Range (V)	Ref
Quasi-2D amorphous Carbon	Top Gate	4	CVD ML-MoS_2	0.7	15.3	95	4	This Work
h-BN	Top Gate	4	Exfoliated MoS_2	~0.7	>30	<93	4	[36]
h-BN	Bottom Gate	8	Exfoliated MoS_2	~0.7	~40	63	6	[37]
h-BN	Top Gate	55	Exfoliated MoS_2	10	33	180	20	[38]
hBN/HfO_2	Bottom Gate	10	CVD ML-MoS_2	0.65	6.8	107	4	[39]
SC-hBN/HfO_2	Bottom Gate	10	CVD ML-MoS_2	0.65	11.8	76	4	[39]
Al_2O_3	Top Gate	25	CVD ML-MoS_2	0.8	24	180	14	[40]
Al_2O_3	Top Gate	6–8	CVD ML-MoS_2	0.65	35	114	8	[41]
AlN/ Al_2O_3	Top Gate	6	CVD ML-MoS_2	0.65	3.3	150	10	[42]
HfO_2	Top Gate	30	CVD ML-MoS_2	0.8	7.8	150	10	[43]
HfO_2	Top Gate	20	CVD ML-MoS_2	0.65	3	130	10	[44]
HfO_2/MgO	Top Gate	>30	Exfoliated MoS_2	~2	26	110	10	[45]
HfO_2/Al_2O_3	Top Gate	>30	Exfoliated MoS_2	~2	50.8	120	20	[45]
HfO_2/Y_2O_3	Top Gate	>30	Exfoliated MoS_2	~2	63.7	100	20	[45]
Perovskite strontium-titanium-oxide	Bottom Gate	30nm	CVD ML-MoS_2	0.7	39.7	71.5	1.5	[46]
CaF_2	Bottom Gate	2nm	CVD 2L-MoS_2	~1.4	NR	90	3.5	[47]

Supplementary Table 3| Comparison of the performances of 2D MoS_2 transistors built with quasi-2D amorphous carbon films, h-BN, or bulk metal oxides as the gate dielectrics. SC: single crystal; ML: monolayer.

Device Structure	Ag/Quasi-2D amorphous Carbon/Pt	Au/h-BN/Cu	Ag/BNO_x/Graphene	Au/MoS_2/Au	Cu/GO/Pt	W/a-CO_x/Pt	TiN/HfO_2/Pt	Al/N-AlOx/Al	Pt/Ta_2O_{5-x}-TaO_{2-x}/Pt	Pt/TiO2/TIN
V_{Form} (V)	Forming free	Forming free	0.66	Forming free	Forming free	5.5	-4	Forming free	~2	5
V_{Set} (V)	0.25	2.70	0.61	0.55	0.6	5	-2.5	2.03	-1	-1.1
V_{Reset} (V)	-0.09	-0.9	-0.25	-0.53	-0.4	-4	2.8	-1.32	2	1
Variability (σ)	0.03/ 0.04	0.25/ 0.16	0.06	0.06/ 0.08	0.2/0.1	NR	0.4/ 0.3	0.3/0.2	NR	NR
Device size	100/200 nm	750 nm	60 nm	15 µm	100 nm	100 nm	20nm	1 µm	30 nm	20 µm
Thickness	1.2-1.5 nm	~2 nm	~0.9 nm	0.7 nm	20 nm	18 nm	5 nm	15 nm	30 nm	80 nm
Endurance	$>10^4$	~100	>100	~150	>100	$>10^4$	$>10^8$	$>10^5$	$>10^{10}$	$>10^6$
On/off Ratio	5×10^4	$\sim10^6$	~500	>100	~50	$\sim10^4$	>10	~100	>10	~100
Retention test (s)	$>10^4$ at 85 ºC	$>10^4$ at RT	$>10^4$ at 85 ºC	$\sim10^6$ at RT	$>10^4$ at RT	$>10^4$ at 85 ºC	$>10^5$ at 125 ºC	$>10^5$ at 125 ºC	$>10^9$ at 85 ºC	$>10^6$ at 85 ºC
Switching Speed t_{Set}	<20 ns	~200 ns	NR	NR	NR	<100 ns	~50 ns	NR	10 ns	5 ns
Energy Consumption	~20 fJ	20 fJ	NR	NR	NR	~5 pJ	~10 pJ	NR	~1 pJ	~10 pJ
Reference	This work	[48]	[49]	[50]	[51]	[52]	[53]	[54]	[55]	[56]

Supplementary Table 4| Performance comparison of memristors based on low-dimensional materials or bulk oxides. BNO_x: oxidized h-BN; GO: graphene oxide; a-CO_x: bulk oxygenated amorphous carbon; NR: not reported; RT: room temperature.

Supplementary References

1 Koh, Y. K., Bae, M.-H., Cahill, D. G. & Pop, E. Reliably counting atomic planes of few-layer graphene (n > 4). *ACS Nano* **5**, 269-274 (2011).

2 Petrova, E., Tinchev, S. & Nikolova, P. Interference effects on the I_D/I_G ratio of the Raman spectra of diamond-like carbon thin films. *arXiv:1112.0897 [cond-mat.mtrl-sci]* (2011).

3 Zhao, X.-M., Wilbur, J. L. & Whitesides, G. M. Using two-stage chemical amplification to determine the density of defects in self-assembled monolayers of alkanethiolates on gold. *Langmuir* **12**, 3257-3264 (1996).

4 Schultz, M. J. *et al.* Synthesis of linked carbon monolayers: Films, balloons, tubes, and pleated sheets. *Proc. Natl. Acad. Sci. U.S.A.* **105**, 7353 (2008).

5 Zhang, Y. *et al.* Investigation of the defect density in ultra-thin Al_2O_3 films grown using atomic layer deposition. *Surf. Coat. Technol.* **205**, 3334-3339 (2011).

6 Shi, Y. *et al.* Synthesis of few-layer hexagonal boron nitride thin film by chemical vapor deposition. *Nano Lett.* **10**, 4134-4139 (2010).

7 Hod, O. Graphite and hexagonal boron-nitride have the same interlayer distance. Why? *J. Chem. Theory Comput.* **8**, 1360-1369 (2012).

8 Song, L. *et al.* Large scale growth and characterization of atomic hexagonal boron nitride layers. *Nano Lett.* **10**, 3209-3215 (2010).

9 Falin, A. *et al.* Mechanical properties of atomically thin boron nitride and the role of interlayer interactions. *Nat. Commun.* **8**, 15815 (2017).

10 Cassabois, G., Valvin, P. & Gil, B. Hexagonal boron nitride is an indirect bandgap semiconductor. *Nat. Photonics* **10**, 262-266 (2016).

11 Elias, C. *et al.* Direct band-gap crossover in epitaxial monolayer boron nitride. *Nat. Commun.* **10**, 2639 (2019).

12 Laturia, A., Van de Put, M. L. & Vandenberghe, W. G. Dielectric properties of hexagonal boron nitride and transition metal dichalcogenides: from monolayer to bulk. *npj 2D Mater. Appl.* **2**, 6 (2018).

13 Ahmed, F. *et al.* Dielectric dispersion and high field response of multilayer hexagonal boron nitride. *Adv. Funct. Mater.* **28**, 1804235 (2018).

14 Britnell, L. *et al.* Electron tunneling through ultrathin boron nitride crystalline barriers. *Nano Lett.* **12**, 1707-1710 (2012).

15 Lee, G.-H. *et al.* Electron tunneling through atomically flat and ultrathin hexagonal boron nitride. *Appl. Phys. Lett.* **99**, 243114 (2011).

16 Ranjan, A. *et al.* Dielectric breakdown in single-crystal hexagonal boron nitride. *ACS Appl. Electron. Mater.* **3**, 3547-3554 (2021).

17 Kim, K. K. *et al.* Synthesis and characterization of hexagonal boron nitride film as a dielectric layer for graphene devices. *ACS Nano* **6**, 8583-8590 (2012).

18 Kim, S. M. *et al.* Synthesis of large-area multilayer hexagonal boron nitride for high material performance. *Nat. Commun.* **6**, 8662 (2015).

19 Jang, S. K., Youn, J., Song, Y. J. & Lee, S. Synthesis and characterization of hexagonal boron nitride as a gate dielectric. *Sci. Rep.* **6**, 30449 (2016).

20 Uddin, M. R., Majety, S., Li, J., Lin, J. Y. & Jiang, H. X. Layer-structured hexagonal (BN)C semiconductor alloys with tunable optical and electrical properties. *J. Appl. Phys.* **115**, 093509 (2014).

21 Doan, T. C. *et al.* Hexagonal boron nitride thin film thermal neutron detectors with high energy resolution of the reaction products. *Nucl. Instrum. Methods Phys. Res., Sect. A* **783**, 121-127 (2015).

22 Kim, S. *et al.* Realization of a high mobility dual-gated graphene field-effect transistor with Al_2O_3 dielectric. *Appl. Phys. Lett.* **94**, 062107 (2009).

23 Xia, F., Perebeinos, V., Lin, Y.-m., Wu, Y. & Avouris, P. The origins and limits of metal–graphene junction resistance. *Nat. Nanotechnol.* **6**, 179-184 (2011).

24 Xia, J., Chen, F., Li, J. & Tao, N. Measurement of the quantum capacitance of graphene. *Nat. Nanotechnol.* **4**, 505-509 (2009).

25 Lee, J. *et al.* High-performance current saturating graphene field-effect transistor with hexagonal boron nitride dielectric on flexible polymeric substrates. *IEEE Electron Device Lett.* **34**, 172-174 (2013).

26 Meric, I. *et al.* Graphene field-effect transistors based on boron–nitride dielectrics. *Proc. IEEE* **101**, 1609-1619 (2013).

27 Lu, G. *et al.* Synthesis of large single-crystal hexagonal boron nitride grains on Cu–Ni alloy. *Nat. Commun.* **6**, 6160 (2015).

28 Nayfeh, O. M. *et al.* Increased mobility for layer-by-layer transferred chemical vapor deposited graphene/boron-nitride thin films. *Appl. Phys. Lett.* **102**, 103115 (2013).

29 Wang, M. *et al.* A platform for large-scale graphene electronics – CVD growth of single-layer graphene on CVD-grown hexagonal boron nitride. *Adv. Mater.* **25**, 2746-2752 (2013).

30 Lee, K. H. *et al.* Large-scale synthesis of high-quality hexagonal boron nitride nanosheets for large-area graphene electronics. *Nano Lett.* **12**, 714-718 (2012).

31 Iqbal, M. W., Iqbal, M. Z., Jin, X., Eom, J. & Hwang, C. Superior characteristics of graphene field effect transistor enclosed by chemical-vapor-deposition-grown hexagonal boron nitride. *J. Mater. Chem. C* **2**, 7776-7784 (2014).

32 Gannett, W. *et al.* Boron nitride substrates for high mobility chemical vapor deposited graphene. *Appl. Phys. Lett.* **98**, 242105 (2011).

33 Lee, C. *et al.* Anomalous lattice vibrations of single- and few-layer MoS_2. *ACS Nano* **4**, 2695-2700 (2010).

34 Mouri, S., Miyauchi, Y. & Matsuda, K. Tunable photoluminescence of monolayer MoS_2 via chemical doping. *Nano Lett.* **13**, 5944-5948 (2013).

35 Ganta, D., Sinha, S. & Haasch, R. T. 2-D material molybdenum disulfide analyzed by XPS. *Surf. Sci. Spectra* **21**, 19-27 (2014).

36 Xie, L. *et al.* Graphene-contacted ultrashort channel monolayer MoS_2 transistors. *Adv. Mater.* **29**, 1702522 (2017).

37 Vu, Q. A. *et al.* Near-zero hysteresis and near-ideal subthreshold swing in h-BN encapsulated single-layer MoS_2 field-effect transistors. *2D Mater.* **5**, 031001 (2018).

38 Roy, T. *et al.* Field-effect transistors built from all two-dimensional material components. *ACS Nano* **8**, 6259-6264 (2014).

39 Chen, T.-A. *et al.* Wafer-scale single-crystal hexagonal boron nitride monolayers on Cu (111). *Nature* **579**, 219-223 (2020).

40 Sanne, A. *et al.* Top-gated chemical vapor deposited MoS_2 field-effect transistors on Si_3N_4 substrates. *Appl. Phys. Lett.* **106**, 062101 (2015).

41 Li, W. *et al.* High-performance CVD MoS_2 transistors with self-aligned top-gate and Bi contact. *Tech. Digest of IEDM* 37.33.31-37.33.34 (2021).

42 Qian, Q. *et al.* Improved gate dielectric deposition and enhanced electrical stability for single-layer MoS_2 MOSFET with an AlN interfacial layer. *Sci. Rep.* **6**, 27676 (2016).

43 Xu, H. *et al.* High-performance wafer-scale MoS_2 transistors toward practical application. *Small* **14**, 1803465 (2018).

44 Wang, L. *et al.* Electronic devices and circuits based on wafer-scale polycrystalline monolayer MoS_2 by chemical vapor deposition. *Adv. Electron. Mater.* **5**, 1900393 (2019).

45 Zou, X. *et al.* Interface engineering for high-performance top-gated MoS_2 field-effect transistors. *Adv. Mater.* **26**, 6255-6261 (2014).

46 Huang, J.-K. *et al.* High-κ perovskite membranes as insulators for two-dimensional transistors. *Nature* **605**, 262-267 (2022).

47 Illarionov, Y. Y. *et al.* Ultrathin calcium fluoride insulators for two-dimensional field-effect transistors. *Nat. Electron.* **2**, 230-235 (2019).

48 Chen, S. *et al.* Wafer-scale integration of two-dimensional materials in high-density memristive crossbar arrays for artificial neural networks. *Nat. Electron.* **3**, 638-645 (2020).

49 Dong, Z. *et al.* Atomically thin CBRAM enabled by 2-D materials: scaling behaviors and performance limits. *IEEE Trans. Electron Devices* **65**, 4160-4166 (2018).

50 Ge, R. *et al.* Atomristor: nonvolatile resistance switching in atomic sheets of transition metal dichalcogenides. *Nano Lett.* **18**, 434-441 (2018).

51 He, C. L. *et al.* Nonvolatile resistive switching in graphene oxide thin films. *Appl. Phys. Lett.* **95**, 232101 (2009).

52 Santini, C. A. *et al.* Oxygenated amorphous carbon for resistive memory applications. *Nat. Commun.* **6**, 8600 (2015).

53 Chen, H. *et al.* HfO_x based vertical resistive random access memory for cost-effective 3D cross-point architecture without cell selector. *IEDM Tech. Digest*, 20.27.21-20.27.24 (2012).

54 Kim, W. *et al.* Forming-free nitrogen-doped AlO_x RRAM with sub-μA programming current. *VLSI Symp. Tech. Digest*, 22-23 (2011).

55 Lee, M.-J. *et al.* A fast, high-endurance and scalable non-volatile memory device made from asymmetric Ta_2O_{5-x}/TaO_{2-x} bilayer structures. *Nat. Mater.* **10**, 625-630 (2011).

56 Yoshida, C., Tsunoda, K., Noshiro, H. & Sugiyama, Y. High speed resistive switching in $Pt/TiO_2/TiN$ film for nonvolatile memory application. *Appl. Phys. Lett.* **91**, 223510 (2007).
