# Supplementary material for: Ultrathin quasi-2D amorphous carbon dielectric prepared from solution precursor for nanoelectronics
Source: Commun Eng. 2023 Dec 20;2:93. doi: 10.1038/s44172-023-00141-9 (PMC10955813; doi:10.1038/s44172-023-00141-9)
Supplement: Supplementary file 2 — Description of Additional Supplementary Files [file 44172_2023_141_MOESM2_ESM.pdf]

Description of Additional Supplementary Files

File name: Supplementary Data 1

Description: Coordinates of structures used in DFT simulations.
